# Broadening symptom criteria improves early case identification in SARS-CoV-2 contacts

**DOI:** 10.1183/13993003.02308-2021

**Published:** 2022-07-07

**Authors:** Hamish Houston, Seran Hakki, Timesh D. Pillay, Kieran Madon, Nieves Derqui-Fernandez, Aleksandra Koycheva, Anika Singanayagam, Joe Fenn, Rhia Kundu, Emily Conibear, Robert Varro, Jessica Cutajar, Valerie Quinn, Lulu Wang, Janakan S. Narean, Mica R. Tolosa-Wright, Jack Barnett, Onn Min Kon, Richard Tedder, Graham Taylor, Maria Zambon, Neil Ferguson, Jake Dunning, Jonathan J. Deeks, Ajit Lalvani

**Affiliations:** 1 NIHR Health Protection Research Unit in Respiratory Infections, Imperial College London, London, UK; 2 National Infection Service, Public Health England, London, UK; 3 Tuberculosis Service, Imperial College Healthcare NHS Trust, London, UK; 4 Molecular Diagnostics Unit, Imperial College London, London, UK; 5 Section of Virology, Dept of Infectious Disease, Imperial College London, London, UK; 6 Dept of Infectious Disease Epidemiology, Faculty of Medicine, Imperial College London, London, UK; 7 NIHR Health Protection Research Unit in Emerging and Zoonotic Infections, University of Oxford, Oxford, UK; 8 Test Evaluation Research Group, Institute of Applied Health Research, University of Birmingham, Birmingham, UK; 9 H. Houston and S. Hakki contributed equally; 10 J.J. Deeks and A. Lalvani contributed equally

## Abstract

**Background:**

The success of case isolation and contact tracing for the control of severe acute respiratory syndrome coronavirus 2 (SARS-CoV-2) transmission depends on the accuracy and speed of case identification. We assessed whether inclusion of additional symptoms alongside three canonical symptoms (CS), *i.e.* fever, cough and loss or change in smell or taste, could improve case definitions and accelerate case identification in SARS-CoV-2 contacts.

**Methods:**

Two prospective longitudinal London (UK)-based cohorts of community SARS-CoV-2 contacts, recruited within 5 days of exposure, provided independent training and test datasets. Infected and uninfected contacts completed daily symptom diaries from the earliest possible time-points. Diagnostic information gained by adding symptoms to the CS was quantified using likelihood ratios and area under the receiver operating characteristic curve. Improvements in sensitivity and time to detection were compared with penalties in terms of specificity and number needed to test.

**Results:**

Of 529 contacts within two cohorts, 164 (31%) developed PCR-confirmed infection and 365 (69%) remained uninfected. In the training dataset (n=168), 29% of infected contacts did not report the CS. Four symptoms (sore throat, muscle aches, headache and appetite loss) were identified as early-predictors (EP) which added diagnostic value to the CS. The broadened symptom criterion “≥1 of the CS, or ≥2 of the EP” identified PCR-positive contacts in the test dataset on average 2 days earlier after exposure (p=0.07) than “≥1 of the CS”, with only modest reduction in specificity (5.7%).

**Conclusions:**

Broadening symptom criteria to include individuals with at least two of muscle aches, headache, appetite loss and sore throat identifies more infections and reduces time to detection, providing greater opportunities to prevent SARS-CoV-2 transmission.

## Abstract 2

**Data from two prospective longitudinal community-based cohorts show broadened symptom criteria identify more SARS-CoV-2 infections and reduce time to detection with minimal reductions in specificity, providing greater opportunities to prevent transmission.** https://bit.ly/3wRpMeW

## Introduction

Severe acute respiratory syndrome coronavirus 2 (SARS-CoV-2) transmission is not entirely prevented by current vaccines [1]. As vaccination coverage increases, blanket isolation rules for SARS-CoV-2 contacts (*i.e.* test–trace–isolate) become less acceptable to society. Rapid identification and isolation of contacts who become infected is an increasingly important alternative strategy for prevention and containment [2]. Effectiveness depends crucially on how quickly such cases are detected and initiate self-isolation [3], because individuals are most infectious early in the course of infection [4, 5]. However, case definitions must also be sufficiently specific to avoid overwhelming testing capacity [6, 7].

There is considerable international heterogeneity in policy for coronavirus disease 2019 (COVID-19) community testing within the general population (supplementary material S1) [8–12]. Most criteria include fever, cough and loss or change in smell or taste (hereafter referred to as the canonical symptoms (CS)) alongside a range of other symptoms. Some countries are currently considering altering their case definitions [13]. There is thus an urgent need for empirical data to identify whether additional symptoms (which we call early-predictors (EP)) can augment the CS within community case definitions.

Surprisingly, empirical longitudinal data from recently exposed SARS-CoV-2 contacts are scarce. Recent large-scale cross-sectional studies of community testing data support adding more symptoms to the CS [7, 14]. Descriptive longitudinal retrospective studies of SARS-CoV-2 infections also exist [15, 16]. However, a high-resolution longitudinal evaluation of symptom combinations for differentiating infected SARS-CoV-2 contacts from exposed but uninfected controls has not, to the best of our knowledge, been performed before.

Using data from two prospective longitudinal cohorts of SARS-CoV-2 contacts, we aimed to establish definitively whether broadening symptom criteria beyond the CS can accelerate and improve case detection without weakening specificity. Rapid recruitment following clearly defined exposure enabled optimal symptom criteria to be identified. Daily contemporaneously recorded symptom diaries ensured symptom onset times were recorded and time-savings measured with maximum precision. Through direct study of relevant community-based cohorts we provide generalisable evidence-based criteria for effective case definitions to rapidly identify and isolate infectious cases.

## Methods

### Recruitment and study procedures

INSTINCT (Integrated Network for Surveillance, Trials and Investigations into COVID-19 Transmission) and ATACCC (Assessment of Transmission And Contagiousness of COVID-19 in Contacts) were two community-based cohort studies in which contacts of COVID-19 cases in Greater London in the UK were identified and recruited from 10 May 2020 through 31 March 2021.

Index cases, or contacts identified by the UK contact tracing system (NHS Test and Trace (NTAT)), were referred from Public Health England (PHE). Initially, referrals were also received from the Royal College of General Practitioners Research and Surveillance Centre (RCGP-RSC) network. Contacts referred within 5 days of their index case symptom onset (ISO) and who provided valid informed consent were enrolled within our recruitment capacity until the end of the second pandemic wave in the UK. Ethics approval was granted by the Health Research Authority (REC 20/NW/0231).

In INSTINCT, household contacts living with their index cases were enrolled at home by research nurses (day 0) and visited again on days 7, 14 and 27. Date of ISO was recorded at enrolment and served as a proxy for exposure. Combined nose and throat swabs (CNTS) for reverse transcriptase (RT)-PCR testing and blood samples for serology were taken by research nurses at each visit and an additional CNTS by participants on day 4. Samples were processed at the Molecular Diagnostics Unit, Imperial College London (London, UK). Antibody (IgM and IgG) to SARS-CoV-2 receptor binding domain (anti-RBD) was measured using a two-step double antigen binding assay with recombinant S1 antigen on the solid phase and labelled recombinant RBD as detector in the fluid phase [17]. In ATACCC, household and non-household contacts (*i.e.* not residing with their index) were enrolled. Dates of ISO (household contacts) or exposure event (non-household contacts) were provided by NTAT. After nurse-delivered training, participants self-sampled CNTS daily for 14 consecutive days. SARS-CoV-2 RT-PCR testing was performed at the Virus Reference Dept, PHE Colindale (London, UK).

At enrolment, demographic information was collected and participants recorded the onset date of prior symptoms. After enrolment, participants completed a daily symptom diary which assessed 20 symptoms (supplementary material S2). Loss or change in smell or taste was recorded as one item (hereafter referred to as anosmia).

### Definitions and reference standards

INSTINCT data were used as the training dataset. “Current infection” was set as the target condition and a rigorous composite reference standard was constructed to establish its presence or absence with maximum accuracy [18]. Contacts were assigned to the “infected” group if they were PCR-positive at day 0, 4 or 7. Contacts were assigned to the “uninfected” group if they were PCR-negative and had undetectable SARS-CoV-2 antibodies at all time-points. Participants were excluded if they had no serology results or were PCR-negative at all time-points but had detectable SARS-CoV-2 antibodies at study day 0, 7 or 27.

ATACCC data were used as the test dataset. In this cohort, daily PCR results were available but serological testing was not performed routinely. Contacts were assigned to the “PCR-positive” group if they had a positive PCR result by 7 days after enrolment and to the “PCR-negative” group if all results were negative. Participants who became PCR-positive after study day 7 or had no PCR results were excluded from the analysis. Participants with only one positive PCR result with a high cycle threshold (*C*_t_) value (>28) were excluded to minimise false-positives caused by recent rather than current infection.

**FIGURE 1:**
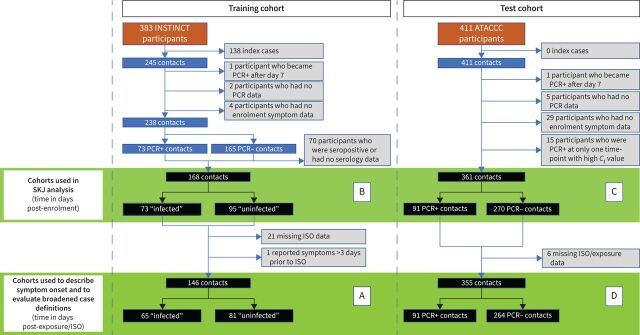
Flow diagram for the inclusion and exclusion of INSTINCT and ATACCC study participants for each analysis. Cohort A was used for a time-to-event analysis describing symptom onset in time post-index case symptom onset (ISO). Cohorts B and C were used to create Spiegelhalter Knill-Jones (SKJ) models for each study day. Cohort D was used to evaluate the performance of simple case definitions at different time-points following exposure. 383 participants were recruited within INSTINCT, of which 138 were indexes and were excluded. Six out of 245 contacts were excluded because of missing PCR data or enrolment symptom data and one was excluded as they became PCR-positive after study day 7. 73 out of 238 contacts were PCR-positive and assigned to the “infected” group. 43 out of 165 PCR-negative contacts were seropositive at study day 0 or 7 (possible prior infection or vaccination), and two out of 165 seroconverted at day 27 (possible separate exposure event) and were excluded. None of the 165 PCR-negative participants seroconverted at study day 14. 25 out of 165 PCR-negative participants had no serology data available and were excluded. 21 out of 168 contacts were excluded from cohort A due to missing ISO date and one was excluded as they reported symptoms several days prior to ISO. 411 contacts were recruited in ATACCC. 34 were excluded because of missing PCR or enrolment symptom data and one was excluded as they became PCR-positive after study day 7. 15 were excluded because they were PCR-positive at only one time-point with a high cycle threshold (*C*_t_) value. 91 of the remaining 361 contacts had at least one positive PCR result by study day 7. Six out of 361 were excluded from cohort D because of missing exposure date.

In both cohorts, participants were made aware of their PCR results as they became available. Participants with missing ISO or exposure dates were excluded from analyses requiring these data. The study flowchart (figure 1) depicts participant numbers included in each analysis.

Results are presented in accordance with STARD (Standards for Reporting of Diagnostic Accuracy Studies) and STROBE (Strengthening Reporting of Observational Studies in Epidemiology) guidelines.

### Statistical analyses

#### Time-to-event analysis

We used time-to-event analysis to describe the onset of COVID-19-related symptoms relative to ISO in INSTINCT (figure 1, cohort A). Briefly, we used symptoms reported by “uninfected” contacts to define baseline time-dependent hazards, and the difference between “infected” and “uninfected” contacts to define COVID-19-related hazards for each symptom (see supplementary material S3 for detailed methodology). Symptoms with a probability of occurring due to COVID-19 of >15% by 10 days post-ISO were selected as candidate symptoms for further evaluation.

#### Spiegelhalter Knill-Jones models

We aimed to quantify any additional diagnostic value gained by adding each of the candidate symptoms to the CS using likelihood ratios (LRs) estimated for individual symptoms within combinations of symptoms. The Spiegelhalter Knill-Jones (SKJ) method was used rather than the independence Bayes approach in order to adjust for dependency caused by symptom co-occurrence [19]. This method is summarised in supplementary material S4, having been described in detail previously [19–23]. Symptoms were considered as a series of binary tests based on their occurrence by each study day (*e.g.* fever by day 3 would be regarded as positive if fever had been reported on study day 2). Persistent cough and productive cough were combined into a single cough variable. We compared models using the CS to those with an additional symptom. The area under the receiver operating characteristic curve (AUC) allows evaluation of model discrimination in training and test datasets. Candidate symptoms with useful LRs after adjustment for dependency with the CS and whose addition improved AUC across multiple early time-points were considered “EP”.

#### Evaluating simple case definitions

To assess real-world impact through readily applicable case definitions, each of the EP was added to the CS individually and together as a list requiring more than one to be positive by using the words “at least”. Diagnostic performance was assessed against the serial PCR reference standard in the test dataset (figure 1, cohort D) at each day post-exposure. We used time-to-event analysis to measure how quickly broadened case definitions would identify PCR-positive individuals and log-rank tests to make comparisons with the CS. Finally, we quantified the prevalence-dependent trade-off between true-positives and false-positives by calculating the number needed to test (NNT): the number of false-positives for every true-positive plus 1.

#### Software

Statistical analyses were performed in Stata version 17.0 (StataCorp, College Station, TX, USA) and R (R Core Team, Vienna, Austria).

## Results

### Patient cohorts

53 011 referrals were received *via* three recruitment pathways (supplementary material S5). Of 529 contacts within two cohorts, 164 (31%) developed PCR-confirmed infection and 365 (69%) remained uninfected. Supplementary material S6 shows demographic details for INSTINCT and ATACCC. Sex, ethnicity and body mass index were similar between cohorts. Participants were slightly older in ATACCC than in INSTINCT (median 38 *versus* 34 years; p<0.001).

Participants were enrolled a median (interquartile range (IQR)) 3 (2–4) days post-ISO in INSTINCT, 4 (4–5) days post-ISO in household contacts in ATACCC and 5 (4–6) days post-exposure event in non-household contacts. In INSTINCT, 22 out of 168 (13.1%) contacts were linked to index cases identified through the RCGP-RSC network with suspected but not confirmed COVID-19 and were included to avoid selection bias. Symptom diaries were completed in INSTINCT for a median (IQR) 26 (7–27) days by “infected” and 7 (0–27) days by “uninfected” contacts, and in ATACCC for 13 (0–20) days by “PCR-positives” and 7 (0–14) days by “PCR-negatives”.

Four participants with confirmed infection required hospitalisation. While >90% of “infected” contacts in INSTINCT (68 out of 73 (93.1%)) reported at least one of the 20 symptoms by day 7, over a quarter (21 out of 73 (28.8%)) did not report fever, cough or anosmia by day 7.

### Sequence of onset of COVID-19-related symptoms

Time-to-event analysis of symptom onset following exposure (supplementary material S7 and S8) showed that fever preceded anosmia and persistent cough preceded productive cough. Sore throat and rhinitis occurred early, and breathlessness later. Fatigue was commonly reported by “uninfected” contacts.

13 symptoms had a probability of occurring due to COVID-19 of >15% by 10 days post-ISO (fever, persistent cough, productive cough, anosmia, headache, muscle aches, sore throat, rhinitis, appetite loss, breathlessness, diarrhoea, nausea and abdominal pain). Nine of these 13 symptoms are not included in the CS and were denoted candidate symptoms in further analyses. Other than the CS; rhinitis, sore throat, headache, muscle aches and appetite loss had the largest cumulative COVID-19-related hazards.

### Additional diagnostic value of candidate symptoms

Raw counts of participants who had reported each symptom by each study day in the training cohort are presented in supplementary material S9. Used alone, cough, rhinitis, headache and muscle aches were the most sensitive symptoms, while nausea and abdominal pain were insensitive (supplementary material S10). Anosmia, fever and appetite loss were highly specific symptoms.

**FIGURE 2:**
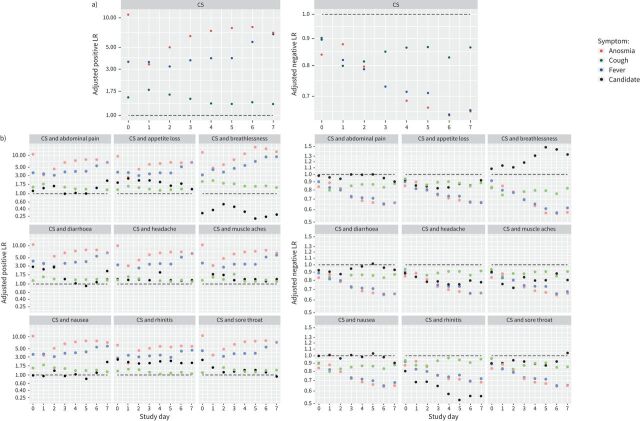
Adjusted likelihood ratios (LRs) for individual symptoms within symptom combinations. Symptoms were considered as a series of binary tests based on their occurrence by each study day. Spiegelhalter Knill-Jones (SKJ) models were created using a) three predictors (fever, cough and anosmia) to evaluate the diagnostic performance of the canonical symptoms (CS) and b) four predictors to evaluate the effect of adding one of the nine candidate variables to the CS (fever, cough, anosmia and candidate). Models were created for the day of enrolment and each of the first 7 study days. Positive and negative LRs for the presence or absence of each symptom by each study day were calculated (supplementary material S11) and then adjusted for dependency with the other predictors within the model to measure the independent predictive value of each symptom within the symptom combination. See supplementary material S4 for a full description of the SKJ method and a worked example. Adjusted LRs for study days 0, 2 and 4 are presented in table 1. Adjusted positive LRs are shown on the left and adjusted negative LRs are shown on the right. In each plot the horizontal line drawn is drawn at 1: LRs above the line increase post-test odds and LRs below the line reduce post-test odds. Bootstrap confidence intervals for adjusted LRs could not be calculated because some bootstrap iterations resulted in samples with singularities.

The crude LRs (supplementary material S11) show that any of the symptoms will affect post-test odds when they are used alone. However, when used in combination with other symptoms, their LRs after adjustment using the SKJ approach (figure 2, table 1 and supplementary material S12) were all less extreme than their crude LRs, indicating considerable dependency between symptoms.

**TABLE 1:** Adjusted likelihood ratios (LRs) for individual symptoms when canonical symptoms (CS) are used in combination with an additional candidate symptom

**SKJ model**	**Study day**	**Symptom**	**Adjusted positive LR**	**Adjusted negative LR**	**SKJ model**	**Study day**	**Symptom**	**Adjusted positive LR**	**Adjusted negative LR**	**SKJ model**	**Study day**	**Symptom**	**Adjusted positive LR**	**Adjusted negative LR**
CS and abdominal pain	0	Fever	3.54	0.90	CS and appetite loss	0	Fever	3.68	0.90	CS and breathlessness	0	Fever	3.10	0.91
		Cough	1.48	0.90			Cough	1.29	0.94			Cough	2.12	0.82
		Anosmia	10.73	0.84			Anosmia	9.46	0.85			Anosmia	11.94	0.83
		Abdominal pain	1.19	0.98			Appetite loss	1.96	0.92			Breathlessness	0.31	1.09
CS and abdominal pain	2	Fever	3.04	0.80	CS and appetite loss	2	Fever	3.00	0.80	CS and breathlessness	2	Fever	3.69	0.76
		Cough	1.62	0.82			Cough	1.44	0.86			Cough	1.88	0.77
		Anosmia	4.42	0.81			Anosmia	4.39	0.81			Anosmia	5.80	0.78
		Abdominal pain	1.58	0.94			Appetite loss	2.22	0.84			Breathlessness	0.52	1.11
CS and abdominal pain	4	Fever	3.83	0.71	CS and appetite loss	4	Fever	3.52	0.73	CS and breathlessness	4	Fever	5.48	0.65
		Cough	1.33	0.87			Cough	1.22	0.90			Cough	1.56	0.80
		Anosmia	7.35	0.69			Anosmia	6.17	0.71			Anosmia	11.10	0.64
		Abdominal pain	1.04	0.99			Appetite loss	2.01	0.83			Breathlessness	0.34	1.31
CS and diarrhoea	0	Fever	4.08	0.89	CS and headache	0	Fever	3.22	0.91	CS and muscle aches	0	Fever	3.64	0.90
		Cough	1.26	0.94			Cough	1.27	0.94			Cough	1.29	0.94
		Anosmia	11.15	0.84			Anosmia	10.29	0.84			Anosmia	10.70	0.84
		Diarrhoea	2.88	0.93			Headache	1.37	0.89			Muscle aches	1.37	0.91
CS and diarrhoea	2	Fever	2.93	0.80	CS and headache	2	Fever	2.69	0.82	CS and muscle aches	2	Fever	2.83	0.81
		Cough	1.38	0.88			Cough	1.44	0.86			Cough	1.35	0.88
		Anosmia	5.43	0.79			Anosmia	4.29	0.81			Anosmia	4.75	0.80
		Diarrhoea	2.81	0.88			Headache	1.33	0.79			Muscle aches	1.76	0.72
CS and diarrhoea	4	Fever	3.82	0.72	CS and headache	4	Fever	3.43	0.73	CS and muscle aches	4	Fever	3.36	0.74
		Cough	1.31	0.87			Cough	1.20	0.91			Cough	1.21	0.91
		Anosmia	7.35	0.69			Anosmia	6.61	0.70			Anosmia	7.05	0.69
		Diarrhoea	1.08	0.98			Headache	1.30	0.76			Muscle aches	1.37	0.80
CS and nausea	0	Fever	3.54	0.90	CS and rhinitis	0	Fever	2.75	0.92	CS and sore throat	0	Fever	2.75	0.92
		Cough	1.53	0.89			Cough	1.28	0.94			Cough	1.28	0.94
		Anosmia	10.86	0.84			Anosmia	6.17	0.87			Anosmia	6.17	0.87
		Nausea	1.02	1.00			Rhinitis	2.57	0.80			Sore throat	2.57	0.80
CS and nausea	2	Fever	3.01	0.80	CS and rhinitis	2	Fever	2.95	0.80	CS and sore throat	2	Fever	2.95	0.80
		Cough	1.56	0.83			Cough	1.40	0.87			Cough	1.40	0.87
		Anosmia	5.08	0.79			Anosmia	3.07	0.85			Anosmia	3.07	0.85
		Nausea	1.30	0.97			Rhinitis	1.99	0.69			Sore throat	1.99	0.69
CS and nausea	4	Fever	3.81	0.72	CS and rhinitis	4	Fever	3.33	0.74	CS and sore throat	4	Fever	3.33	0.74
		Cough	1.31	0.87			Cough	1.06	0.97			Cough	1.06	0.97
		Anosmia	7.36	0.69			Anosmia	5.58	0.72			Anosmia	5.58	0.72
		Nausea	1.08	0.99			Rhinitis	2.29	0.58			Sore throat	2.29	0.58

LRs for individual symptoms in combination with other symptoms were calculated using the Spiegelhalter Knill-Jones (SKJ) method which adjusts for the dependency caused by symptom co-occurrence. Adjusted LRs for study days 0–7 are represented graphically in figure 2 and values for study days 0, 2 and 4 are presented here. For any combination of symptoms at a particular time-point, the adjusted LRs can be multiplied together to estimate effect on post-test odds. For example, on study day 0, a contact with fever and headache but without cough or anosmia has 3.22×0.94×0.84×1.37=3.5 times the odds of being infected compared with a contact where symptoms are unknown (see supplementary material S4 for a worked example).

When cough was used in combination with fever and anosmia its adjusted LRs were closer to 1 than those of anosmia or fever (figure 2a and supplementary material S12). This was most likely due to the higher specificity of anosmia and fever (supplementary material S10). When combined with the CS, the presence or absence of nausea did not independently affect post-test odds, with its adjusted LRs lying close to 1 (figure 2b). Breathlessness was more common in the “infected” group. However, while breathlessness was reported without fever, anosmia or cough by “uninfected” contacts, this was rare in “infected” contacts, explaining why its adjusted positive LRs are <1 and negative LRs are >1.

**FIGURE 3:**
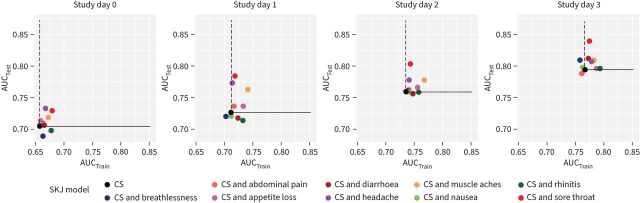
Direct comparison of the area under the receiver operating characteristic curve (AUC) in training and test datasets. Symptoms were considered as a series of binary tests based on their occurrence by each study day. A series of Spiegelhalter Knill-Jones (SKJ) models were created, one for each study day, using three predictors (fever, cough and anosmia) to evaluate the canonical symptoms (CS) (black data points). Nine further series of models were created using four predictors (fever, cough, anosmia and candidate) to evaluate the effect of adding one of the nine candidate variables to the CS at each study day (coloured data points). Model predictions were evaluated in training and test datasets by calculating the AUC. AUC_Train_: AUC in training dataset (figure 1, cohort B). AUC_Test_: AUC in test dataset (figure 1, cohort C). The solid line marks AUC_Test_ for the CS model. The dashed line marks AUC_Train_ for the CS model. Models where the addition of a candidate symptom yielded better predictions in the training dataset lie to the right of the dashed line and models where better predictions were yielded in the test dataset lie above the solid line.

In training and test datasets, AUCs increased with study day, reflecting improved discrimination afforded by greater accumulation of symptoms by later study days in “infected” contacts (figure 3). AUC was often greater in test data than training data, likely reflecting the longer median time to recruitment in ATACCC.

Between study days 0 and 3, the addition of headache, sore throat, muscle aches and appetite loss to the CS yielded the greatest improvements in AUC in the test dataset. When combined with the CS, appetite loss, headache, sore throat and muscle aches all consistently had positive adjusted LRs >1 and negative adjusted LRs <1, showing that both their presence and their absence added to the CS's ability to discriminate between the infected and uninfected. These symptoms were therefore considered “EP”.

### Evaluating simple case definitions

Each of the four EP were combined individually with the CS using an “OR” operator, as well as together using “OR” and “AT LEAST” operators (box 1).

**FIGURE 4:**
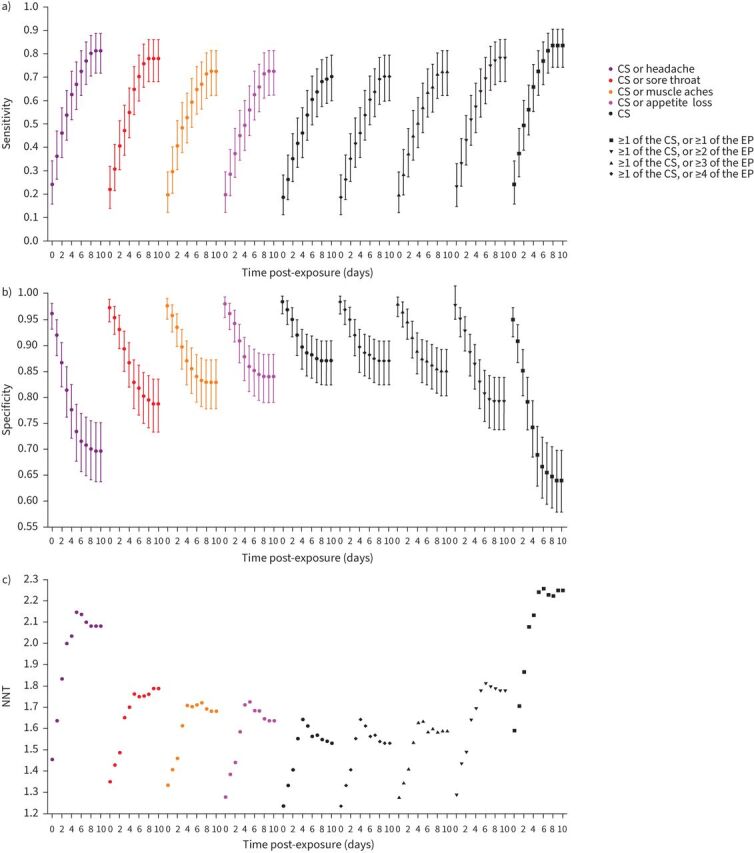
a) Sensitivity, b) specificity and c) number needed to test (NNT) for the canonical symptoms (CS) and broadened symptom criteria. The early-predictors (EP) (sore throat, headache, muscle aches and appetite loss) were each combined individually with the CS (fever, cough and anosmia) using an “OR” operator and all were added together using “AT LEAST” and “OR” operators (as described in box 1). Sensitivity, specificity and NNT were calculated for each symptom criterion by day post-exposure (index case symptom onset for household contacts) against a serial PCR reference standard. Full results are given in supplementary material S13. NNT is calculated by dividing the number of false-positives by the number of true-positives and adding 1. Rarely, symptoms were reported at enrolment without an onset date. We imputed onset dates for these symptoms by assuming the median number of days pre-enrolment (maximum two participants (0.55%) for rhinitis).

The addition of any symptom to the CS using an “OR” operator increased sensitivity (figure 4a and supplementary material S13) while reducing specificity (figure 4b and supplementary material S13). The addition of appetite loss produced the smallest changes compared with the CS.

**BOX 1:** Construction of easily comprehensible case definitions using Boolean operators

Case definitions can contain Boolean logical operators such as AND, OR and AT LEAST (see supplementary material S1 for examples of international case definitions in current use)	
**Symptom groups**	**Referred to in text as**
fever, cough, anosmia	Canonical symptoms (CS)
headache, sore throat, muscle aches, appetite loss	Early-predictors (EP)
**The EP were each combined individually with the CS using an “OR” operator**	**Referred to in text as**
fever OR cough OR anosmia OR headache	CS or headache
fever OR cough OR anosmia OR sore throat	CS or sore throat
fever OR cough OR anosmia OR muscle aches	CS or muscle aches
fever OR cough OR anosmia OR appetite loss	CS or appetite loss
**The EP were all combined together with the CS using “AT LEAST” and “OR” operators**	**Referred to in text as**
fever OR cough OR anosmia OR AT LEAST 1 of headache, sore throat, muscle aches and appetite loss	≥1 of the CS, or ≥1 of the EP
fever OR cough OR anosmia OR AT LEAST 2 of headache, sore throat, muscle aches and appetite loss	≥1 of the CS, or ≥2 of the EP
fever OR cough OR anosmia OR AT LEAST 3 of headache, sore throat, muscle aches and appetite loss	≥1 of the CS, or ≥3 of the EP
fever OR cough OR anosmia OR AT LEAST 4 of headache, sore throat, muscle aches and appetite loss	≥1 of the CS, or ≥4 of the EP

**FIGURE 5:**
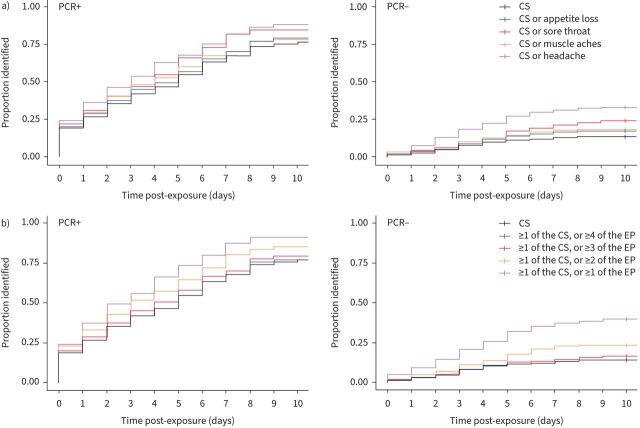
Kaplan–Meier plots showing time from exposure until positive identification by the canonical symptoms (CS) and broadened symptom criteria. The early-predictors (EP) (sore throat, headache, muscle aches and appetite loss) were a) each combined individually with the CS using an “OR” operator and b) all were added together using “AT LEAST” and “OR” operators (as described in box 1). The proportion of PCR-positive (left) and PCR-negative (right) participants who were positively identified by each case definition by each day following exposure (index case symptom onset for household contacts) is shown using a Kaplan–Meier plot. The plot for the CS is shown in black. Life-tables are presented in supplementary material S14 and median time to diagnosis in table 2. Rarely, symptoms were reported at enrolment without an onset date. We imputed onset dates for these symptoms by assuming the median number of days pre-enrolment (maximum two participants (0.55%) for rhinitis).

The CS identified 50% of PCR-positives by 6 days post-exposure (figure 5, table 2 and supplementary material S14). Adding headache yielded the greatest increase in sensitivity (figure 4a and supplementary material S13) and would identify PCR-positives on average 2 days earlier (p=0.02), but causes the largest reduction in specificity (15.2% at 5 days post-exposure) (figure 4b and supplementary material S13). In contrast, “CS or sore throat” only reduced specificity by 5.7% at 5 days and identified PCR-positive cases earlier than the CS, by 1 day on average. This change was not statistically significant given the small number of PCR-positive participants in the cohort (p=0.1, n=91).

**TABLE 2:** Time until positive identification by simple case definitions including the canonical symptoms (CS) and broadened symptom criteria

**Case definition**	**PCR-positive**			**PCR-negative**		
	**Time until 25% identified (days post-exposure)**	**Time until 50% identified (days post-exposure)**	**Time until 75% identified (days post-exposure)**	**Time until 25% identified (days post-exposure)**	**Time until 50% identified (days post-exposure)**	**Time until 75% identified (days post-exposure)**
**CS**	2	6	10	Never	Never	Never
**CS or sore throat**	2	5	8	Never	Never	Never
**CS or headache**	2	4	7	6	Never	Never
**CS or muscle aches**	2	5	9	Never	Never	Never
**CS or appetite loss**	2	6	9	Never	Never	Never
**≥1 of the CS, or ≥1 of the EP**	2	4	7	5	Never	Never
**≥1 of the CS, or ≥2 of the EP**	2	4	8	Never	Never	Never
**≥1 of the CS, or ≥3 of the EP**	2	5	9	Never	Never	Never
**≥1 of the CS, or ≥4 of the EP**	2	6	9	Never	Never	Never

EP: early-predictors. Contacts were assigned to the “PCR-positive” group if they had a positive PCR result by 7 days after enrolment and to the “PCR-negative” group if all results were negative. The EP (sore throat, headache, muscle aches and appetite loss) were each combined individually with the CS using an “OR” operator and all were added together using “AT LEAST” and “OR” operators (as described in box 1).

When all four EP are added to the CS, if all four are required, there is very little difference to the CS. In contrast, the case definition “≥1 of the CS, or ≥1 of the EP” would increase sensitivity and identify PCR-positive cases a median 2 days earlier than the CS (p=0.002). However, the corresponding reduction in specificity by 5 days post-exposure (19.7%) (figure 4b and supplementary material S13) would lead to 25% of PCR-negative individuals being inappropriately identified (table 2 and figure 5b). “≥1 of the CS, or ≥2 of the EP” identified PCR-positive cases a median 2 days earlier than the CS (p=0.07) with a reduction in specificity of only 5.7% at 5 days post-exposure. This reduction is smaller than that caused by moving from the CS to various other international case definitions (supplementary material S15). None of the EP were dispensable from this proposed criterion (supplementary material S16).

The number of individuals identified in order to yield a single PCR-positive case, *i.e.* the NNT, increases rapidly immediately after exposure, reflecting an initial accumulation of false-positives because no one has yet developed symptoms actually caused by infection (figure 4c). NNT plateaus ∼4–5 days following exposure, reflecting the incubation period. At 25.6% prevalence, “≥1 of the CS, or ≥2 of the EP” had a NNT at 5 days post-exposure of 1.78 compared with 1.61 for the CS, indicating 17 additional individuals identified for every 100 infected individuals identified.

## Discussion

To the best of our knowledge, this is the first study to use daily symptom data prospectively collected from recently exposed infected and uninfected SARS-CoV-2 contacts to evaluate the diagnostic performance of symptom combinations for detecting infection.

Using this definitive study design, we found that 29% of individuals with PCR-confirmed COVID-19 did not report any of the CS, but 93% reported at least one symptom from a broader list of 20. We identified four EP symptoms (sore throat, headache, muscle aches and appetite loss) providing additional early predictive power for identifying SARS-CoV-2-infected contacts. The case definition “≥1 of the CS, or ≥2 of the EP” identified PCR-positive contacts 2 days earlier after exposure than the CS alone (p=0.07). This time-saving is critical given that shortening the delay from infectiousness to self-isolation from 2.6 to 1.2 days has been estimated to reduce transmission by 47% [3]. Moreover, the proportion of “symptomatic” infections and time to symptom onset are critical parameters in studies modelling effectiveness of testing and isolation strategies for contacts [24].

Consistent with previous studies, headache and sore throat were sensitive symptoms [7], which occurred early in the course of infection [15] and were prevalent in our relatively young participants [25]. The importance of these symptoms will increase as vaccination of older age groups increases the proportion of infections occurring in the young. In agreement with the Real-time Assessment of Community Transmission-1 (REACT-1) study, we found that headache, muscle aches and appetite loss improved discrimination within statistical prediction models [14]. We add a crucial evaluation of readily applicable case definitions. We observed that both the structure of symptom criteria (*e.g.* use of the Boolean operator “AT LEAST”) and time from exposure had a considerable effect on diagnostic performance.

The SKJ approach enabled another important new observation. Although an important indicator of disease severity [26], breathlessness was not a useful additional symptom for identifying early and mild infections because a hierarchy of symptoms exists. Breathlessness is unlikely to occur due to COVID-19 without prior fever, cough or anosmia and its inclusion reduces specificity.

Further strengths include day-by-day measurement of diagnostic performance following exposure and prospective data collection which mitigates recall bias. The rigorous reference standard employed in our training cohort maximised accuracy for the target condition and ensured only the most useful symptoms were taken forward to the test data. Neither serology nor PCR have 100% sensitivity for SARS-CoV-2 infection [27]. Using both serology and PCR at multiple time-points to define the absence of infection, we minimised false-negatives. False-positive PCR results caused by recent rather than current infection were likely less common in our longitudinal study of recently exposed contacts than in studies involving random community sampling [14].

Limitations include modest sample size, largely White British population, minor differences between training and test cohorts, and the potential for tick-box and behavioural biases. Study participants were usually highly motivated, and attentiveness to mild symptoms (*e.g.* rhinitis) may have been increased by awareness of exposure, frequent study visits and co-residence with other participants. Contacts could not be blinded to their PCR results or those of their index.

Since we studied community-based COVID-19 contacts identified through NTAT, our findings are very likely generalisable. As large-scale cross-sectional data replicate our findings in smaller-scale daily-resolution longitudinal data, the combined evidence base is now sufficient to influence policy. Broadening symptom criteria for use in the general population would likely identify more infections and reduce time to detection, reducing transmission. We propose that symptom criteria within case definitions to prompt symptomatic isolation and testing of SARS-CoV-2 contacts should include headache, sore throat, muscle aches and appetite loss as well as the CS to optimise sensitivity. Two of these additional symptoms should be required to maximise specificity.

As highly vaccinated regions transition to lower COVID-19 incidence, investment in RT-PCR testing capacity will make such broader case definitions feasible. As societies develop alternatives to test–trace–isolate, application of evidence-based symptom criteria alongside judicious testing will be critical for early discrimination of infected and uninfected contacts. Accordingly, our findings should inform development of evidence-based national testing policies in many parts of the world now and in subsequent phases of the pandemic.

## Supplementary material

10.1183/13993003.02308-2021.Supp1 **Please note:** supplementary material is not edited by the Editorial Office, and is uploaded as it has been supplied by the author.
Supplementary material ERJ-02308-2021.Supplement

## Shareable PDF

10.1183/13993003.02308-2021.Shareable1 This one-page PDF can be shared freely online.
Shareable PDF ERJ-02308-2021.Shareable
