# Supplementary material for: Broadening symptom criteria improves early case identification in SARS-CoV-2 contacts
Source: Eur Respir J. 2022 Jul 7;60(1):2102308. doi: 10.1183/13993003.02308-2021 (PMC8620106; doi:10.1183/13993003.02308-2021)

SUPPLEMENTARY MATERIALS:

S1: International Variation in Symptom Criteria within Case Definitions for suspected COVID-19 within the general community population (as of 16th July 2021):

**World Health Organisation**	*1)* Fever *AND* Cough<br>*2) AT LEAST 3 OF:* Fever, Cough, Fatigue, Headache, Myalgia, Sore throat, Coryza, Anorexia/nausea/vomiting, Dyspnoea, Diarrhoea, Altered mental status<br>*3)* Loss or change [illegible] Anorexia/Nausea/Vomiting [illegible]*bsence of any other identified cause*
**United States (Center for Disease Control)**	*1)* Cough *OR* Dyspnoea *OR* Shortness of breath *OR* Loss or change in smell or taste<br>*2) AT LEAST 2 OF:* Fever, Chills, Rigors, Myalgia, Headache, Sore throat, Diarrhoea, Fatigue, Coryza, Nausea/vomiting
**France**	Dyspnoea *OR* Cough *OR* Fever *OR* Loss or change in smell or taste<br>Fatigue *OR* Sore throat *OR* Headache *OR* Myalgia
**Spain**	Dyspnoea *OR* Cough *OR* Fever *OR* Loss or change in smell or taste<br>Diarrhoea *OR* Sore throat *OR* Headache *OR* Myalgia
**United Kingdom**	Cough *OR* Fever *OR* Loss or change in smell or taste

Exact wordings of criteria can be found at: World Health Organization,[8] United States (Centers for Disease Control),[9] France,10] Spain,[11] United Kingdom.[12]

SUPPLEMENTARY METHODS:

S2: Precise Wording of Symptom Items within Symptom Diary. The order in which items were asked about within the symptom diary is given in the right-hand column.

Type	Referred to in main text as	Wording within symptom diary<br>In the last 24 hours, have you…	Order within symptom diary
Canonical Symptom	fever	felt feverish?	1
	persistent cough	had a persistent cough lasting for more than 4 hours?	2
	productive cough	coughed up phlegm or mucous?	3
	anosmia	had loss or change of smell or taste?	17
Lower Respiratory	breathlessness	felt unusually dyspnoea?	5
	wheeze	had a wheeze?	20
	cough blood	coughed blood?	4
	chest pain	had chest pain?	11
Upper Respiratory	rhinitis	had a runny or blocked nose?	14
	sore throat	had a sore throat?	13
	conjunctivitis	had red or irritated eyes?	16
Gastrointestinal	abdominal pain	had unusual tummy pains?	19
	appetite loss	had loss of appetite?	18
	nausea	felt sick or vomited?	7
	diarrhoea	had diarrhoea?	10
Systemic	headache	had a headache?	12
	muscle aches	had unusual muscle aches?	6
	fatigue	felt tired or generally unwell?	8
	confusion	been confused?	9
	rash	had a rash?	15

S3: Describing symptom onset using time-to-event analysis

We aimed to identify symptoms with a high probability of occurring due to COVID-19 by 10 days post index symptom onset. Index symptom onset (ISO) was defined as the onset date of the first of any of the 20 symptoms. We used time-to-event analysis to describe the onset of COVID-19 related symptoms by comparing symptom onset in 'infected' and 'uninfected' contacts in cohort A (Figure 1).

Participants were excluded from this analysis if the date of index symptom onset was unknown, or if the contact reported symptoms which occurred more than 3 days prior to index symptom onset. Rarely, symptoms were reported at enrolment without an onset date. We imputed onset dates for these symptoms using the median number of days pre-enrolment (maximum 9 participants [5.4%] for muscle aches). Participants with incomplete symptom diaries were right-censored on the day their symptom diary ended.

We used symptoms reported by 'uninfected' contacts to define a baseline time-dependent hazard and calculated the difference in hazards between 'infected' and 'uninfected' contacts to represent the COVID-19-related time-dependent hazard of onset of each symptom.

$$h(t) = h^{+}(t) - h^{-}(t)$$

Cumulative COVID-19-related hazards $H(t)$ were calculated by adding up the daily $\mathrm{h}(\mathrm{t})$ up to time t. The probability of an 'infected' individual experiencing a symptom as a result of their infection by a particular time t, was estimated by calculating 1 minus the survival probability $S(t)$:

$$1 - S(t) = 1 - (\exp[-H(t)]).$$

We selected symptoms where this probability was more than 15% at $t = 10$ days as candidate symptoms for further evaluation. Bootstrap 90% confidence intervals were calculated for h(t) and H(t).

S4: Spiegelhalter Knill-Jones Method

Likelihood ratios (LRs) have clinical value as they quantify how much a diagnostic test result (or symptom) will raise or lower the pre-test probability of the target condition. Bayes's theorem (by which LRs are calculated) assumes that diagnostic tests act independently. However, symptoms often co-occur and this dependency must be accounted for to avoid overoptimistic evaluations of diagnostic performance. Spiegelhalter and Knill-Jones combined Bayes's theorem with logistic regression to produce LRs which are adjusted for dependency.

We used Spiegelhalter Knill-Jones models to study the predictive power of symptom combinations and measure the diagnostic usefulness of each symptom within the combinations. The analysis was performed using Cohort B as the training dataset and Cohort C as the test dataset (Figure 1).

Symptoms were considered as diagnostic tests based on their occurrence by each study day for the day of recruitment (day 0) and the first 7 days thereafter. Diagnostic performance is assessed compared to a composite serial PCR and serology reference standard in the training dataset and a serial PCR reference standard in the test dataset (described in the main text).

A series of 8 Spieglhalter Knill-Jones models were created, one for each study day, using 3 predictors (fever, cough, anosmia) to evaluate the canonical symptoms (CS). Nine further sets of 8 models were created using 4 predictors (fever, cough, anosmia, and the candidate symptom) to evaluate the effect of adding one of the 9 candidate variables to the 3 CS at each study day. Adjusted LRs were calculated for each of the canonical symptoms (fever, cough and anosmia) when used together, and for each candidate symptom when added to the canonical symptoms.

In brief, rather than including sets of (0,1) indicator variables for each symptom in a logistic regression, the indicator variables take the value of the crude positive and negative likelihood ratios LR^{crude} in a logistic regression equation of the form:

$$\log(posttest\ odds) = \log(pretest\ odds) + \gamma_1 logLR_1^{crude} + \gamma_2 logLR_2^{crude}$$

using log(pretest odds) as an offset term and not including an intercept term. Missing symptom data are entered as having log likelihood ratios of zero (making the assumption that a missing value is not in itself diagnostic). The parameter γ_i is called the shrinkage factor for symptom i. A value of γ_i of 1 indicates no dependency between symptoms in the model. A value of zero indicates that the symptom adds no additional diagnostic information as there is total dependency with the other symptoms. Adjusted LRs for each symptom when used in combination with the other symptoms in the model are calculated using the shrinkage factor and the crude LR for that symptom when used as on its own.

The formula for calculating adjusted LRs is given by

$$LR_i^{adj} = \exp\left(y_i \times \log\left(LR_i^{crude}\right)\right).$$

We calculated the area under the receiver operating characteristic curve (AUC-ROC) for each predictive model in the training dataset and test dataset (cohort B & C respectively, Figure 1) and compared to the AUC-ROCs for predictive models containing only fever, cough and anosmia. Candidate symptoms whose addition yielded consistent improvements in AUC-ROC across multiple early time-points were considered ‘early predictors’ and were evaluated within simple diagnostic decision tools.

Worked example of a Spiegelhalter Knill-Jones models.

A Spiegelhalter Knill-Jones model using fever, cough, anosmia and headache as predictor variables at study day 0 is presented as a worked example to illustrate how the adjusted likelihood ratios (LRs) are calculated and interpreted. The full output of every model is available on request.

A 2x2 table is constructed for each symptom (raw counts from Table 1 in main text):

	infected	uninfected		
	fever			
symptom present	13	2	Total (TP + FP)	15
symptom absent	60	93	Total (TN + FN)	153
	cough			
symptom present	25	10	Total (TP + FP)	35
symptom absent	48	85	Total (TN + FN)	133
	Anosmia			
symptom present	15	1	Total (TP + FP)	16
symptom absent	58	94	Total (TN + FN)	152
	Headache			
symptom present	29	16	Total (TP + FP)	45
symptom absent	44	79	Total (TN + FN)	123
	Total (TP + FN)	Total (TN + FP)		
	73	95		

Crude positive and negative likelihood ratios (LRs) (S11) are calculated for each symptom i using the equations:

$$positive\ LR_i^{crude} = \frac{\left(\frac{TP}{TP+FN}\right)}{1-\left(\frac{TN}{TN+FP}\right)} \qquad negative\ LR_i^{crude} = \frac{1-\left(\frac{TP}{TP+FN}\right)}{\left(\frac{TN}{TN+FP}\right)}$$

	Crude Positive LR	Crude Negative LR
Fever	8.46	0.84
Cough	3.25	0.73
Anosmia	19.52	0.80
Headache	2.36	0.72

The log of the odds of infection at study day 1 is used as the offset term:

$$log\left(\frac{73}{95}\right) = \log(0.77)$$

Instead of 1 and 0, the indicator variables take the value of the crude positive and negative likelihood ratios for each of the CS with missing data entered as having log likelihood ratios of zero. The model fitted for the example is:

$$\log(posttest\ odds) =$$
$$\log(0.77) + 0.547log(LR^{crude}_{fever}) + 0.204log(LR^{crude}_{cough}) + 0.785log(LR^{crude}_{anosmia}) + 0.366log(LR^{crude}_{headache})$$

The shrinkage factor γ_i is used to calculate the adjusted LRs (Figure 2) for symptom i. For example, the positive adjusted LR for fever when used in combination with cough, anosmia & sore throat on day 0 is calculated as follows:

$$positive\ LR^{adj}_{fever} = \exp(y_{fever} \times \log(positive\ LR^{crude}_{fever})) = \exp(0.547 \times \log(8.46)) = 3.22$$

Note how the magnitude of the LR has been shrunk to account for the symptom's dependency with other symptoms in the model.

Symptom	Crude Positive LR	Crude Negative LR	Shrinkage Factor	Adjusted Positive LR	Adjusted Negative LR
Fever	8.46	0.84	0.547	3.22	0.91
Cough	3.25	0.73	0.204	1.27	0.94
Anosmia	19.52	0.80	0.785	10.29	0.84
Headache	2.36	0.72	0.366	1.37	0.89

Estimates of overall LRs for combinations of test results are obtained by multiplying these values. On study day 0, a contact with fever and headache but without cough or anosmia has $3.22 \times 0.94 \times 0.84 \times 1.37 = 3.5$ times the odds of being infected compared to a contact where symptoms are unknown.

We then used AUC-ROC to evaluate model discrimination in training and test datasets (cohorts B & C, Figure 1) and compare models using the CS to those with an additional symptom (Figure3).

SUPPLEMENTARY RESULTS:

S5: Recruitment cascade.

Early in the study, contacts were referred to our study through the Royal College of General Practitioners Research and Surveillance Centre. Later in the study, contacts were referred either directly through National Test And Trace or, in the case of most household-exposed contacts, through their index case, which PHE had identified. In total, 53011 referrals were received via these three pathways. If contacts were identified via PHE, PHE would initially ascertain whether participants consented to being contacted to take part in a research study. Consenting Contacts would then be contacted by Imperial College London, of which a subset would agree to take part. PHE and Imperial strove to contact all eligible contacts, but were limited by staff resources. The potentially eligible contacts PHE and Imperial attempted to call were non-biased in that the staff systematically worked down their list of participants, which was organised at random. Where referrals concerned index cases, multiple contacts may have been recruited following a single referral.

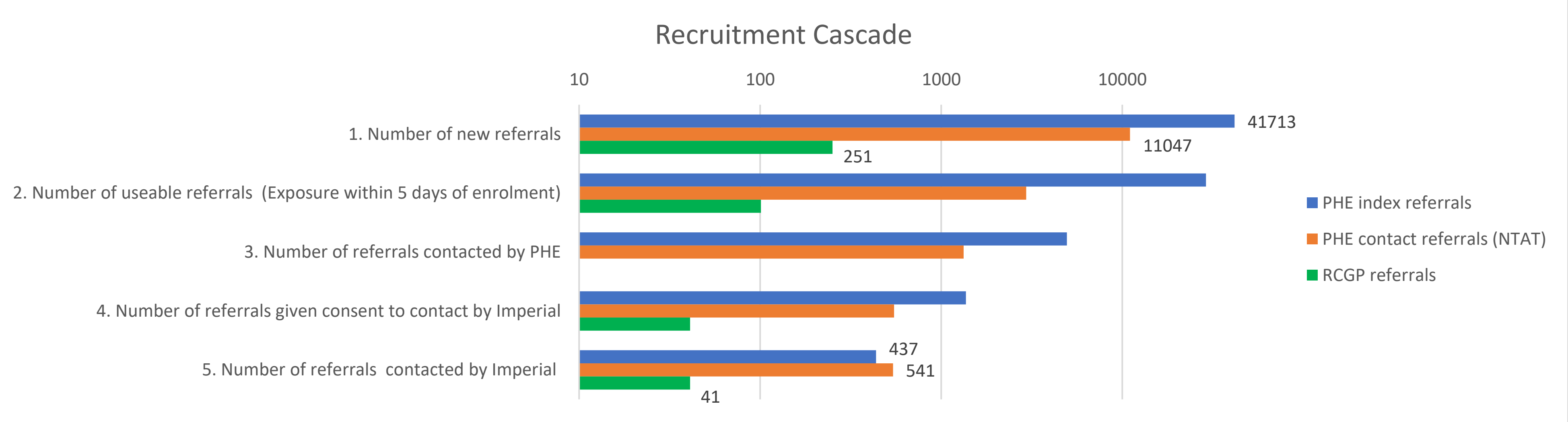

S6: Baseline Demographics for INSTINCT and ATACCC cohorts. Demographics are shown for cohorts B and C in Figure 1. Cohorts were compared using chi-squared tests for proportions and Wilcoxon rank sum to compare non-normally distributed populations.

BMI = Body Mass Index, IQR = Inter-quartile range

		**INSTINCT**	**ATACCC**	**p-value**
N		**168**	**361**	
Contact Type	Household (**n/N**, %)	**168/168**, 100%	**227/361**, 62.9%	**X**
	Non-household (**n/N**, %)	**0/168**, 0%	**134/361**, 37.1%	**X**
Age	(**median**, IQR)	**34**, 21 – 47	**38**, 28 – 52	**<0.001**
Sex	Female (**n/N**, %)	**79/168**, 47.0%	**182/361**, 50.4%	**0.33**
	Male (**n/N**, %)	**88/168**, 52.4%	**166/361**, 46.0%	
BMI	(**median**, IQR)	**25.2**, 22.8 – 27.4	**25.2**, 22.3 – 29.1	**0.93**
Ethnicity	White (**n/N**, %)	**138/168**, 82.1%	**299/361**, 82.8%	**0.93**
	Non-white (**n/N**, %)	**25/168**, 14.9%	**51/361**, 14.1%	
Smoking History	Current smoker (**n/N**, %)	**20/168**, 11.9%	**57/361**, 15.8%	**0.67**
	Vape/E-cigarettes (**n/N**, %)	**4/168**, 2.4%	**8/361**, 2.2%	
	Ex-smoker (**n/N**, %)	**17/168**, 10.1%	**30/361**, 8.3%	
	Non-smoker (**n/N**, %)	**118/168**, 70.2%	**257/361**, 71.2%	
Number of Comorbidities	None (**n/N**, %)	**141/166,** 84.9% (2 missing)	**235/353,** 66.6% (8 missing)	**0.002**
	One or more (**n/N**, %)	**25/166,** 15.1% (2 missing)	**118/353,** 33.4% (8 missing)	

S7: Cumulative COVID-19 related hazards for the onset of each symptom over time in days post index symptom onset. Cumulative COVID-19 related hazards $H(t)$ were calculated by adding up the daily hazards $\mathrm{h(t)}$ up to time t. As $H(t)$ is conditional on not having experienced the symptom before t, for a population it may sum over 1. The probability of an 'infected' individual experiencing a symptom as a result of their infection by a particular time t, was estimated by the formula: $1 - S(t) = 1 - (\exp[-H(t)])$. Symptoms which had $1 - S(t)$ of more than 0.15 at $t = 10days$, corresponding to $H(10days)$ of more than $-\ln(0.85) = 0.163$ were selected as candidate symptoms for evaluation. ISO = Index Symptom Onset.

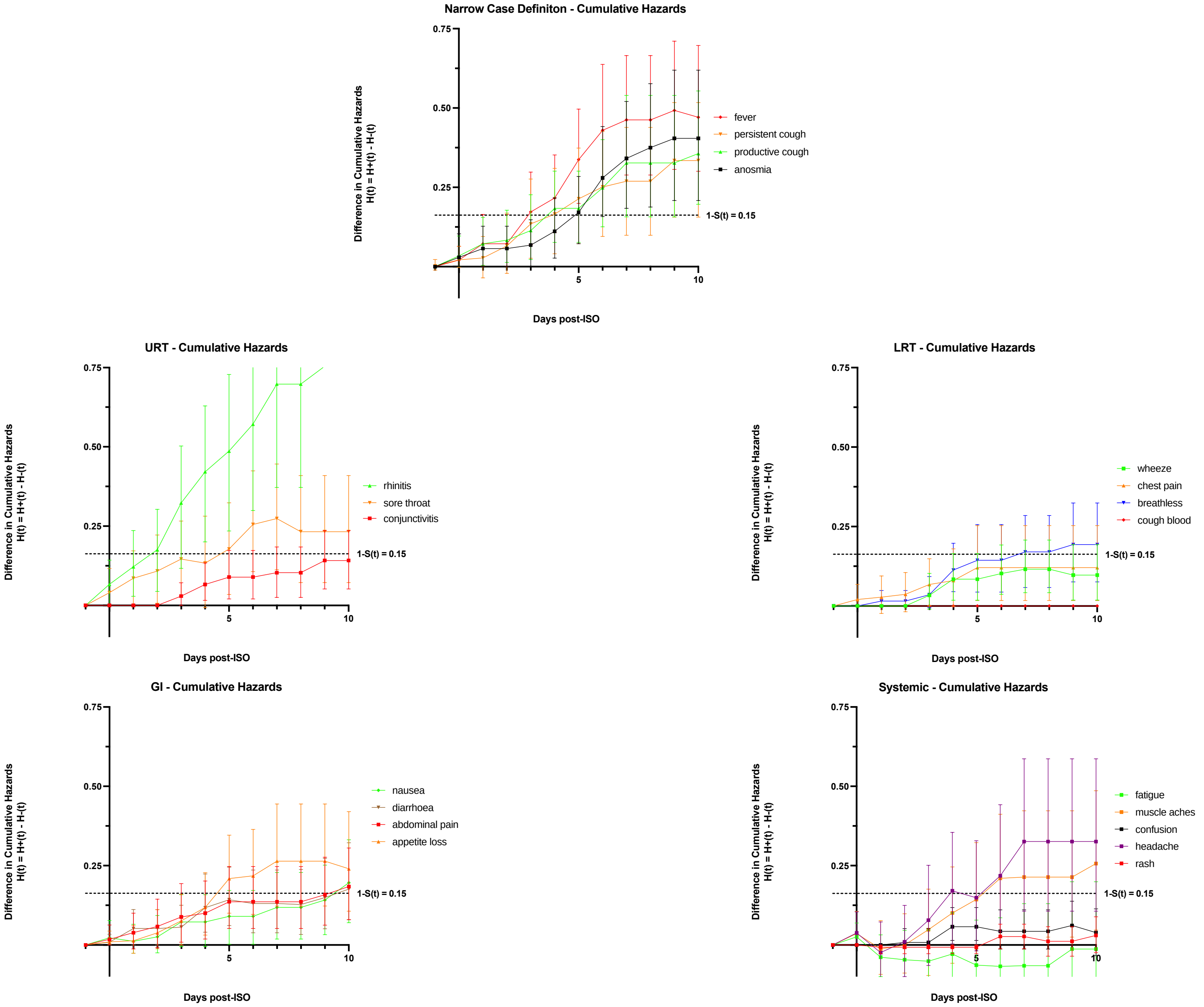

S8: Life table for time-to-event analysis describing time from onset of any index symptom to onset of individual symptoms in the contact. This analysis was performed using Cohort A (Figure 1). For each 2 day time interval, the number of participants at risk of onset of each symptom at the start of each time period is given. The numbers of participants reporting symptom onset, and participants whose study diary ended within the time interval are given in brackets. At enrolment, some contacts reported symptom onset prior to any symptom onset in the index. These events are shown in time periods -4 to -2 and -2 to 0 (days post index symptom onset). As prospective study diaries always started after index symptom onset, the number of participants whose symptom diary ended within these time intervals is not applicable (NA). Rarely, symptoms were reported at enrolment without an onset date. We imputed onset dates for these symptoms using the median number of days pre-enrolment (maximum 9 participants [5.4%] for muscle aches). URT = Upper Respiratory Tract, LRT = Lower Respiratory Tract, GI = Gastrointestinal

			Time Period (Days post index symptom onset)							
			-4 to -2	-2 to 0	0 to 2	2 to 4	4 to 6	6 to 8	8 to 10	10 to 12
Infection Status	Group	Symptom	Number at risk at start of time period (Symptom First reported, Last Day of Symptom Diary)							
Current Infection	Canonical Symptoms	fever		65 (2,NA)	63 (6,0)	57 (10,0)	47 (7,0)	40 (6,1)	33 (1,1)	31 (0,4)
		persistent cough		65 (1,NA)	64 (1,0)	63 (4,3)	56 (8,3)	45 (4,3)	38 (3,4)	31 (3,1)
		productive cough	65 (1,NA)	64 (2,NA)	62 (6,1)	55 (5,0)	50 (4,0)	46 (8,0)	38 (1,0)	37 (2,6)
		anosmia	65 (1,NA)	64 (1,NA)	63 (5,1)	57 (4,0)	53 (5,0)	48 (9,0)	39 (4,1)	34 (1,5)
	URT	rhinitis	65 (2,NA)	63 (2,NA)	61 (10,1)	50 (12,0)	38 (8,0)	30 (8,0)	22 (3,1)	18 (1,2)
		sore throat	65 (1,NA)	64 (0,NA)	64 (8,3)	53 (5,0)	48 (4,4)	40 (4,0)	36 (0,5)	31 (0,6)
		conjunctivitis			65 (0,1)	64 (2,0)	62 (4,2)	56 (1,2)	53 (2,3)	48 (1,9)
	LRT	dyspnoea	65 (2,NA)	63 (0,NA)	63 (1,1)	61 (2,0)	59 (7,3)	49 (2,1)	46 (1,2)	43 (1,8)
		wheeze	65 (1,NA)	64 (1,NA)	63 (0,1)	62 (3,0)	59 (3,0)	56 (2,2)	52 (1,4)	47 (0,9)
		chest pain	65 (1,1)	63 (0,NA)	63 (4,0)	59 (4,0)	55 (4,0)	51 (0,2)	49 (1,3)	45 (0,9)
		cough blood			65 (0,1)	64 (0,0)	64 (0,0)	64 (0,3)	61 (0,4)	57 (1,10)
	GI	diarrhoea	65 (1,NA)	64 (0,NA)	64 (2,4)	57 (1,1)	55 (6,0)	49 (0,1)	48 (2,3)	43 (2,8)
		appetite loss	65 (1,1)	63 (1,NA)	62 (2,4)	56 (4,3)	49 (6,1)	42 (4,2)	36 (0,2)	34 (0,9)
		abdominal pain	65 (1,NA)	64 (0,NA)	64 (4,1)	59 (5,0)	54 (3,0)	51 (0,1)	50 (1,4)	45 (1,7)
		nausea	65 (0,1)	64 (0,NA)	64 (2,2)	60 (5,0)	55 (1,3)	51 (2,2)	47 (3,2)	42 (2,8)
	Systemic	muscle aches	65 (2,NA)	63 (0,NA)	63 (4,7)	52 (6,7)	39 (4,4)	31 (3,0)	28 (1,1)	26 (1,6)
		headache	65 (1,NA)	64 (3,2)	59 (4,7)	48 (6,8)	34 (4,6)	24 (5,0)	19 (1,1)	17 (0,1)
		fatigue	65 (2,2)	61 (0,NA)	61 (2,11)	48 (3,11)	34 (2,5)	27 (2,2)	23 (1,2)	20 (1,4)
		confusion			65 (0,1)	64 (2,0)	62 (3,0)	59 (0,2)	57 (1,4)	52 (0,9)
		rash			65 (0,1)	64 (0,0)	64 (0,0)	64 (2,3)	59 (0,3)	56 (1,10)
Uninfected	Canonical Symptoms	fever			81 (1,2)	77 (1,2)	74 (0,14)	60 (1,5)	54 (0,8)	46 (1,10)
		persistent cough		81 (1,NA)	80 (2,3)	75 (2,2)	71 (1,13)	57 (1,5)	51 (0,9)	42 (0,10)
		productive cough			81 (1,2)	77 (3,2)	72 (1,14)	57 (0,5)	52 (0,9)	43 (0,11)
		anosmia			81 (0,3)	78 (1,2)	75 (0,14)	61 (0,5)	56 (1,9)	46 (0,11)
	URT	rhinitis			81 (4,3)	74 (1,2)	71 (5,14)	52 (1,5)	46 (0,9)	37 (0,9)
		sore throat		81 (1,NA)	80 (2,3)	75 (2,2)	71 (4,14)	53 (0,5)	48 (2,8)	38 (0,9)
		conjunctivitis			81 (0,3)	78 (0,2)	76 (1,15)	60 (0,5)	55 (0,8)	47 (0,11)
	LRT	dyspnoea			81 (0,4)	77 (2,2)	73 (2,15)	56 (2,5)	49 (0,6)	43 (0,10)
		wheeze			81 (0,3)	78 (1,2)	75 (0,14)	61 (0,5)	56 (1,9)	46 (0,12)
		chest pain			81 (2,3)	76 (1,2)	73 (2,14)	57 (0,6)	51 (0,8)	43 (0,9)
		cough blood			81 (0,3)	78 (0,2)	76 (0,14)	62 (0,5)	57 (0,9)	48 (0,12)
	GI	diarrhoea			81 (0,3)	78 (1,2)	75 (2,14)	59 (1,5)	53 (1,9	43 (0,11)
		appetite loss			81 (1,3)	77 (1,2)	74 (1,14)	60 (1,6)	53 (0,8)	45 (1,11)
		abdominal pain			81 (1,3)	77 (1,2)	74 (1,14)	59 (0,5)	54 (0,8)	46 (0,11)
		nausea			81 (1,3)	77 (1,2)	74 (0,15)	59 (0,5)	54 (0,8)	46 (0,11)
	Systemic	muscle aches			81 (5,5)	71 (3,2)	65 (3,15)	51 (3,6)	45 (0,6)	38 (0,8)
		headache			81 (6,4)	71 (3,2)	66 (3,15)	48 (3,6)	39 (0,6)	33 (0,8)
		fatigue			81 (5,3)	73 (6,3)	64 (4,14)	46 (4,5)	37 (0,6)	31 (0,7)
		confusion			81 (0,3)	78 (2,2)	74 (0,14)	60 (2,5)	53 (0,8)	45 (1,11)
		rash			81 (1,3)	77 (0,2)	75 (0,14)	61 (0,5)	56 (1,8)	47 (0,11)

S9: Number of participants who had experienced a particular symptom by study day. Symptoms were considered as a series of binary tests based on their occurrence by each study day. Contacts were defined as infected or uninfected based on a composite PCR and serology reference standard (see main text). These raw counts were used to calculate diagnostic sensitivity and specificity of individual symptoms (S10) as well as crude likelihood ratios (S11) which were used in the Spiegelhalter Knill-Jones analysis. TP = True-positive, FN = False-negative, TN = True-negative, FP = False-positive.

Infection Status			fever	cough	anosmia	muscle aches	headache	sore throat	rhinitis	breathless	appetite loss	abdominal pain	nausea	diarrhoea
	Day	TP + FN	TP	TP	TP	TP	TP	TP	TP	TP	TP	TP	TP	TP
Infected	0	73	13	25	15	25	29	14	26	5	15	9	7	9
	1	72	20	31	15	34	39	20	37	11	20	12	10	12
	2	72	24	33	21	39	45	25	40	15	23	14	14	15
	3	71	28	34	26	40	47	27	43	18	26	14	14	15
	4	71	29	39	28	42	49	29	46	20	28	14	16	18
	5	69	28	38	28	41	48	30	48	20	28	14	16	20
	6	65	29	40	28	38	46	30	46	20	27	14	16	22
	7	57	25	33	25	35	39	26	40	16	23	12	15	18
	Day	TN + FP	FP	FP	FP	FP	FP	FP	FP	FP	FP	FP	FP	FP
Uninfected	0	95	2	10	1	14	16	5	7	6	4	5	4	3
	1	62	3	9	2	11	16	7	10	5	4	4	4	3
	2	62	4	10	2	13	18	10	11	5	5	4	4	3
	3	61	4	9	2	15	19	10	11	7	5	5	4	5
	4	61	4	10	2	15	20	11	11	8	5	5	4	6
	5	58	4	10	2	14	19	11	11	8	5	5	4	6
	6	57	3	10	2	13	18	11	11	7	4	4	3	6
	7	55	2	9	2	12	17	12	11	6	5	3	2	5

S10: Sensitivity and specificity, of individual symptoms by study day. Symptoms were considered as a series of binary tests based on their presence by each study day. Raw counts are given in S9. Diagnostic sensitivity [A] and specificity [B]) were calculated compared to a composite PCR and serology reference standard (described in the main text) in cohort B (Figure 1). The Clopper-Pearson interval was used to calculate 95% confidence intervals.

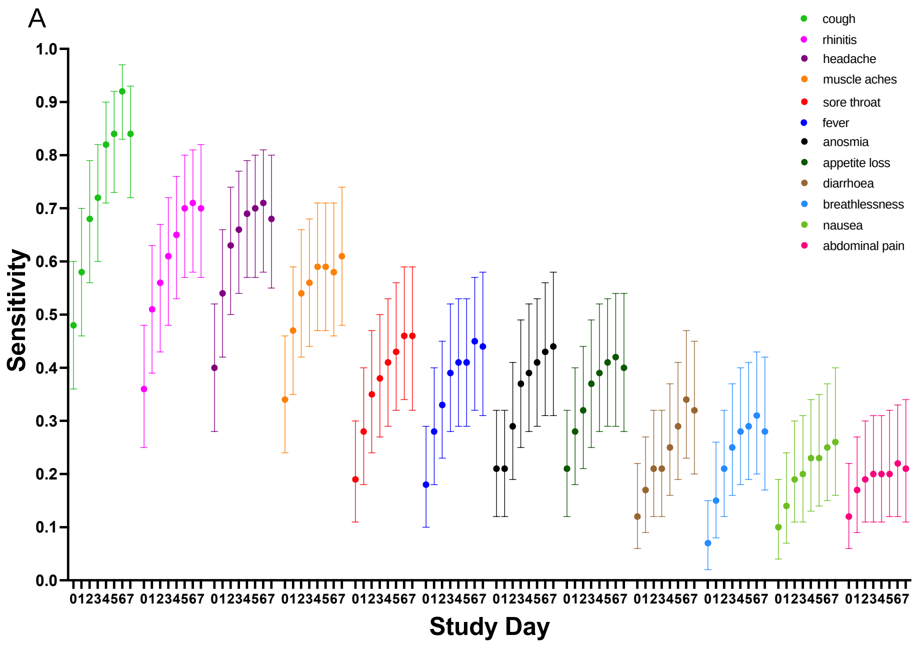

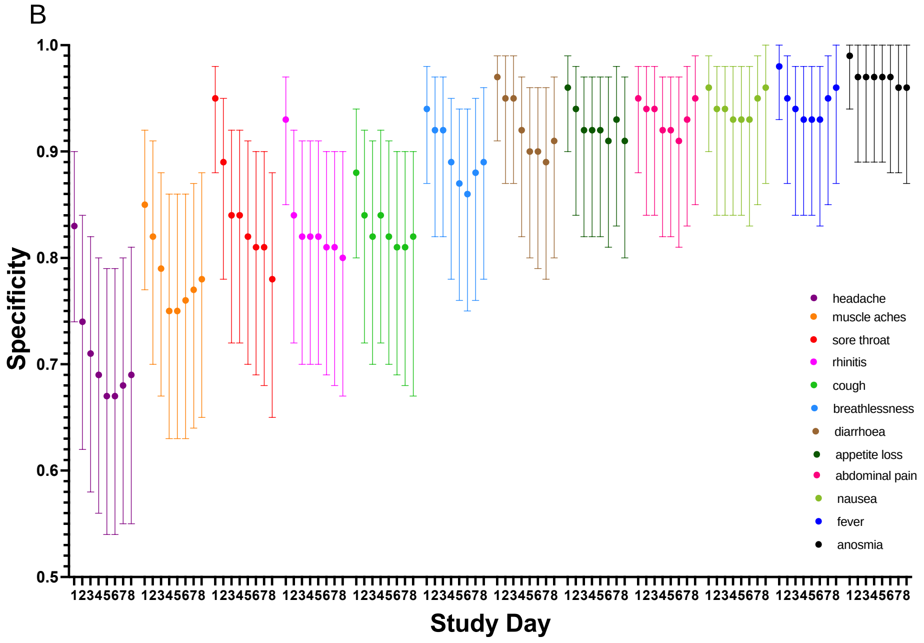

S11: Positive and Negative Crude Likelihood Ratios for individual symptoms. Symptoms were considered as a series of binary tests based on their presence by each study day. Raw counts are given in S9. Crude LRs were calculated compared to a composite PCR and serology reference standard (described in the main text) in cohort B (Figure 1). The Koopman method was used to calculate 95% confidence intervals. LR = likelihood ratio.

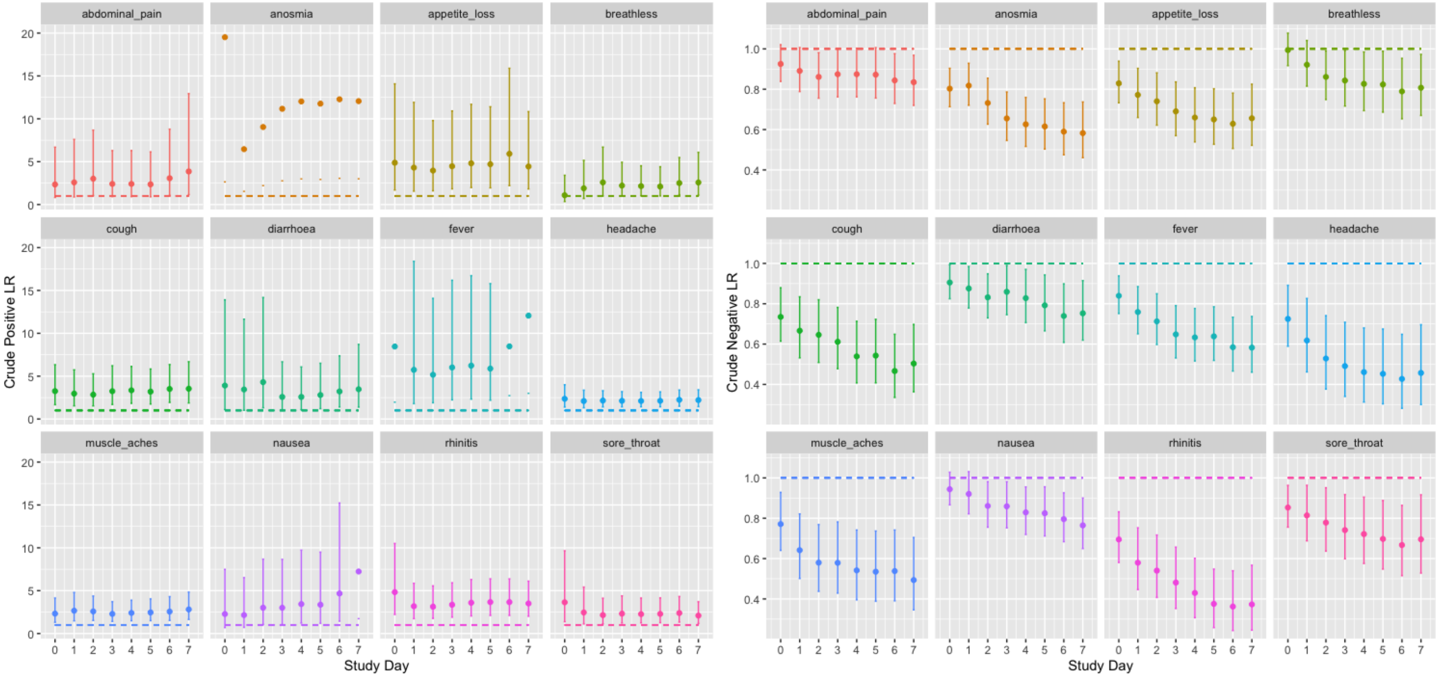

S12: Adjusted Likelihood Ratios for the canonical symptoms when used in combination. LR = likelihood ratio.

Study Day	Symptom	Adjusted Positive LR	Adjusted Negative LR	Study Day	Symptom	Adjusted Positive LR	Adjusted Negative LR
0	fever	3.55	0.9	4	fever	3.87	0.71
	cough	1.53	0.89		cough	1.33	0.86
	anosmia	10.84	0.84		anosmia	7.38	0.69
1	fever	3.53	0.82	5	fever	3.87	0.71
	cough	1.83	0.8		cough	1.31	0.87
	anosmia	3.36	0.88		anosmia	7.87	0.67
2	fever	3.18	0.79	6	fever	5.67	0.65
	cough	1.63	0.81		cough	1.36	0.83
	anosmia	4.97	0.8		anosmia	8.06	0.64
3	fever	3.68	0.73	7	fever	6.83	0.66
	cough	1.48	0.85		cough	1.31	0.86
	anosmia	6.53	0.72		anosmia	7.04	0.65

S13 (overleaf): Sensitivity, specificity and Number-Needed-to-Test by day since exposure for broadened case definitions. Participants were excluded from this analysis if the date of exposure (index symptom onset for household contacts) was unknown The 4 'early predictor' (EP) symptoms (muscle aches, headache, sore throat, appetite loss) were combined individually with the canonical symptoms (CS) using an "OR" operator, for example *fever OR cough OR anosmia OR headache* (S13A), as well as together in a list using an "at least" operator, for example *fever OR cough OR anosmia OR more than 2 of muscle aches, headache, sore throat, appetite loss* (S13B) as described in Box 1 (main text). Diagnostic performance was evaluated for each case definition against a serial PCR reference standard in the test dataset. The increase in TP and FP compared to the CS (*fever OR cough OR* anosmia) was measured. Number needed to test was calculated (number of false positives for every true positive plus 1). The Clopper-Pearson interval was used to calculate confidence intervals for proportions.Click or tap here to enter text. The data in this table are visualised in figure 4 in the main text. TP = true positive, FN = false negative, TN = true negative, FP = false positive, c.f. = compared to, Pos LR = positive likelihood ratio, Neg LR = Negative likelihood ratio, upper.ci = upper 95% confidence interval, lower.ci = lower 95% confidence interval.

S13A: "OR rule" decision tools

Decision Tool	Days post exposure	TP	FN	Increase in TP	Sensitivity	upper.ci	lower.ci	TN	FP	Increase in FP	Specificity	upper.ci	lower.ci	NNT
fever OR cough OR anosmia (CS)	0	17	74	NA	0.19	0.28	0.11	260	4	NA	0.98	1.00	0.96	1.24
	1	24	67	NA	0.26	0.37	0.18	256	8	NA	0.97	0.99	0.94	1.33
	2	32	59	NA	0.35	0.46	0.25	251	13	NA	0.95	0.97	0.92	1.41
	3	38	53	NA	0.42	0.53	0.32	243	21	NA	0.92	0.95	0.88	1.55
	4	42	49	NA	0.46	0.57	0.36	237	27	NA	0.90	0.93	0.85	1.64
	5	49	42	NA	0.54	0.64	0.43	234	30	NA	0.89	0.92	0.84	1.61
	6	55	36	NA	0.60	0.71	0.50	233	31	NA	0.88	0.92	0.84	1.56
	7	58	33	NA	0.64	0.74	0.53	231	33	NA	0.88	0.91	0.83	1.57
	8	62	29	NA	0.68	0.78	0.58	230	34	NA	0.87	0.91	0.82	1.55
	9	63	28	NA	0.69	0.78	0.59	230	34	NA	0.87	0.91	0.82	1.54
	10	64	27	NA	0.70	0.79	0.60	230	34	NA	0.87	0.91	0.82	1.53
CS or muscle aches	0	18	73	1	0.20	0.29	0.12	258	6	2	0.98	0.99	0.95	1.33
	1	27	64	3	0.30	0.40	0.21	253	11	3	0.96	0.98	0.93	1.41
	2	37	54	5	0.41	0.51	0.30	247	17	4	0.94	0.96	0.90	1.46
	3	44	47	6	0.48	0.59	0.38	237	27	6	0.90	0.93	0.85	1.61
	4	48	43	6	0.53	0.63	0.42	230	34	7	0.87	0.91	0.82	1.71
	5	54	37	5	0.59	0.70	0.49	226	38	8	0.86	0.90	0.81	1.70
	6	59	32	4	0.65	0.75	0.54	222	42	11	0.84	0.88	0.79	1.71
	7	61	30	3	0.67	0.77	0.56	220	44	11	0.83	0.88	0.78	1.72
	8	65	26	3	0.71	0.80	0.61	219	45	11	0.83	0.87	0.78	1.69
	9	66	25	3	0.73	0.81	0.62	219	45	11	0.83	0.87	0.78	1.68
	10	66	25	2	0.73	0.81	0.62	219	45	11	0.83	0.87	0.78	1.68
CS or headache	0	22	69	5	0.24	0.34	0.16	254	10	6	0.96	0.98	0.93	1.45
	1	33	58	9	0.36	0.47	0.26	243	21	13	0.92	0.95	0.88	1.64
	2	42	49	10	0.46	0.57	0.36	229	35	22	0.87	0.91	0.82	1.83
	3	49	42	11	0.54	0.64	0.43	215	49	28	0.81	0.86	0.76	2.00
	4	57	34	15	0.63	0.73	0.52	205	59	32	0.78	0.83	0.72	2.04
	5	61	30	12	0.67	0.77	0.56	194	70	40	0.73	0.79	0.68	2.15
	6	66	25	11	0.73	0.81	0.62	189	75	44	0.72	0.77	0.66	2.14
	7	70	21	12	0.77	0.85	0.67	187	77	44	0.71	0.76	0.65	2.10
	8	73	18	11	0.80	0.88	0.71	185	79	45	0.70	0.76	0.64	2.08
	9	74	17	11	0.81	0.89	0.72	184	80	46	0.70	0.75	0.64	2.08
	10	74	17	10	0.81	0.89	0.72	184	80	46	0.70	0.75	0.64	2.08
CS or sore throat	0	20	71	3	0.22	0.32	0.14	257	7	3	0.97	0.99	0.95	1.35
	1	28	63	4	0.31	0.41	0.22	252	12	4	0.95	0.98	0.92	1.43
	2	37	54	5	0.41	0.51	0.30	246	18	5	0.93	0.96	0.89	1.49
	3	43	48	5	0.47	0.58	0.37	236	28	7	0.89	0.93	0.85	1.65
	4	50	41	8	0.55	0.65	0.44	229	35	8	0.87	0.91	0.82	1.70
	5	59	32	10	0.65	0.75	0.54	219	45	15	0.83	0.87	0.78	1.76
	6	64	27	9	0.70	0.79	0.60	216	48	17	0.82	0.86	0.77	1.75
	7	69	22	11	0.76	0.84	0.66	212	52	19	0.80	0.85	0.75	1.75
	8	71	20	9	0.78	0.86	0.68	210	54	20	0.80	0.84	0.74	1.76
	9	71	20	8	0.78	0.86	0.68	208	56	22	0.79	0.84	0.73	1.79
	10	71	20	7	0.78	0.86	0.68	208	56	22	0.79	0.84	0.73	1.79
CS or appetite loss	0	18	73	1	0.20	0.29	0.12	259	5	1	0.98	0.99	0.96	1.28
	1	26	65	2	0.29	0.39	0.20	254	10	2	0.96	0.98	0.93	1.38
	2	34	57	2	0.37	0.48	0.27	249	15	2	0.94	0.97	0.91	1.44
	3	41	50	3	0.45	0.56	0.35	240	24	3	0.91	0.94	0.87	1.59
	4	45	46	3	0.49	0.60	0.39	232	32	5	0.88	0.92	0.83	1.71
	5	51	40	2	0.56	0.66	0.45	227	37	7	0.86	0.90	0.81	1.73
	6	57	34	2	0.63	0.73	0.52	225	39	8	0.85	0.89	0.80	1.68
	7	60	31	2	0.66	0.76	0.55	223	41	8	0.84	0.89	0.80	1.68
	8	65	26	3	0.71	0.80	0.61	222	42	8	0.84	0.88	0.79	1.65
	9	66	25	3	0.73	0.81	0.62	222	42	8	0.84	0.88	0.79	1.64
	10	66	25	2	0.73	0.81	0.62	222	42	8	0.84	0.88	0.79	1.64

S13B: “At least” decision tools

Decision Tool	Days post exposure	TP	FN	Increase in TP	Sensitivity	upper.ci	lower.ci	TN	FP	Increase in FP	Specificity	upper.ci	lower.ci	NNT
fever OR cough OR anosmia (CS)	0	17	74	NA	0.19	0.28	0.11	260	4	NA	0.98	1.00	0.96	1.24
	1	24	67	NA	0.26	0.37	0.18	256	8	NA	0.97	0.99	0.94	1.33
	2	32	59	NA	0.35	0.46	0.25	251	13	NA	0.95	0.97	0.92	1.41
	3	38	53	NA	0.42	0.53	0.32	243	21	NA	0.92	0.95	0.88	1.55
	4	42	49	NA	0.46	0.57	0.36	237	27	NA	0.90	0.93	0.85	1.64
	5	49	42	NA	0.54	0.64	0.43	234	30	NA	0.89	0.92	0.84	1.61
	6	55	36	NA	0.60	0.71	0.50	233	31	NA	0.88	0.92	0.84	1.56
	7	58	33	NA	0.64	0.74	0.53	231	33	NA	0.88	0.91	0.83	1.57
	8	62	29	NA	0.68	0.78	0.58	230	34	NA	0.87	0.91	0.82	1.55
	9	63	28	NA	0.69	0.78	0.59	230	34	NA	0.87	0.91	0.82	1.54
	10	64	27	NA	0.70	0.79	0.60	230	34	NA	0.87	0.91	0.82	1.53
≥1 of the CS, or ≥1 of the EP	0	22	69	5	0.24	0.34	0.16	251	13	9	0.95	0.97	0.92	1.59
	1	34	57	10	0.37	0.48	0.27	240	24	16	0.91	0.94	0.87	1.71
	2	45	46	13	0.49	0.60	0.39	225	39	26	0.85	0.89	0.80	1.87
	3	51	40	13	0.56	0.66	0.45	209	55	34	0.79	0.84	0.74	2.08
	4	60	31	18	0.66	0.76	0.55	196	68	41	0.74	0.79	0.69	2.13
	5	66	25	17	0.73	0.81	0.62	182	82	52	0.69	0.74	0.63	2.24
	6	70	21	15	0.77	0.85	0.67	176	88	57	0.67	0.72	0.61	2.26
	7	74	17	16	0.81	0.89	0.72	173	91	58	0.66	0.71	0.59	2.23
	8	76	15	14	0.84	0.90	0.74	171	93	59	0.65	0.71	0.59	2.22
	9	76	15	13	0.84	0.90	0.74	169	95	61	0.64	0.70	0.58	2.25
	10	76	15	12	0.84	0.90	0.74	169	95	61	0.64	0.70	0.58	2.25
≥1 of the CS, or ≥2 of the EP	0	21	70	4	0.23	0.33	0.15	258	6	2	0.98	0.99	0.95	1.29
	1	30	61	6	0.33	0.44	0.23	251	13	5	0.95	0.97	0.92	1.43
	2	39	52	7	0.43	0.54	0.33	245	19	6	0.93	0.96	0.89	1.49
	3	47	44	9	0.52	0.62	0.41	234	30	9	0.89	0.92	0.84	1.64
	4	52	39	10	0.57	0.67	0.46	228	36	9	0.86	0.90	0.82	1.69
	5	58	33	9	0.64	0.74	0.53	219	45	15	0.83	0.87	0.78	1.78
	6	63	28	8	0.69	0.78	0.59	213	51	20	0.81	0.85	0.75	1.81
	7	68	23	10	0.75	0.83	0.65	210	54	21	0.80	0.84	0.74	1.79
	8	70	21	8	0.77	0.85	0.67	209	55	21	0.79	0.84	0.74	1.79
	9	71	20	8	0.78	0.86	0.68	209	55	21	0.79	0.84	0.74	1.77
	10	71	20	7	0.78	0.86	0.68	209	55	21	0.79	0.84	0.74	1.77
≥1 of the CS, or ≥3 of the EP	0	18	73	1	0.20	0.29	0.12	259	5	1	0.98	0.99	0.96	1.28
	1	26	65	2	0.29	0.39	0.20	255	9	1	0.97	0.98	0.94	1.35
	2	34	57	2	0.37	0.48	0.27	250	14	1	0.95	0.97	0.91	1.41
	3	41	50	3	0.45	0.56	0.35	242	22	1	0.92	0.95	0.88	1.54
	4	46	45	4	0.51	0.61	0.40	235	29	2	0.89	0.93	0.85	1.63
	5	52	39	3	0.57	0.67	0.46	231	33	3	0.88	0.91	0.83	1.63
	6	58	33	3	0.64	0.74	0.53	230	34	3	0.87	0.91	0.82	1.59
	7	60	31	2	0.66	0.76	0.55	228	36	3	0.86	0.90	0.82	1.60
	8	65	26	3	0.71	0.80	0.61	226	38	4	0.86	0.90	0.81	1.58
	9	66	25	3	0.73	0.81	0.62	225	39	5	0.85	0.89	0.80	1.59
	10	66	25	2	0.73	0.81	0.62	225	39	5	0.85	0.89	0.80	1.59
≥1 of the CS, or ≥4 of the EP	0	17	74	0	0.19	0.28	0.11	260	4	0	0.98	1.00	0.96	1.24
	1	24	67	0	0.26	0.37	0.18	256	8	0	0.97	0.99	0.94	1.33
	2	32	59	0	0.35	0.46	0.25	251	13	0	0.95	0.97	0.92	1.41
	3	38	53	0	0.42	0.53	0.32	243	21	0	0.92	0.95	0.88	1.55
	4	42	49	0	0.46	0.57	0.36	237	27	0	0.90	0.93	0.85	1.64
	5	49	42	0	0.54	0.64	0.43	234	30	0	0.89	0.92	0.84	1.61
	6	55	36	0	0.60	0.71	0.50	233	31	0	0.88	0.92	0.84	1.56
	7	58	33	0	0.64	0.74	0.53	231	33	0	0.88	0.91	0.83	1.57
	8	63	28	1	0.69	0.78	0.59	230	34	0	0.87	0.91	0.82	1.54
	9	64	27	1	0.70	0.79	0.60	230	34	0	0.87	0.91	0.82	1.53
	10	64	27	0	0.70	0.79	0.60	230	34	0	0.87	0.91	0.82	1.53

S14 (overleaf): Life-tables for time-to-event analysis describing time in days from exposure until participants were positively identified by a case definition. Easily comprehensible case definitions containing the canonical symptoms (CS): fever, cough and anosmia, and the early-predictors (EP): headache, sore throat, muscle aches and appetite loss were constructed as described in Box 1 (main text).The number of participants to whom the case definition was applied at each time-point is given (‘at risk'). The numbers of participants identified positively by the case definition (‘identified’), and the number of participants whose study diary ended (‘lost’) are given for each time-point. The median, 25th and 75th percentile of the time till positive identification by each case definition in days post exposure is given for PCR-positive and PCR-negative participants (also shown in Table 2, main text). Where participants were identified at enrolment, this value is 0 representing study day 0. If 25% of participants were not identified by the case definition, the value is “Never”. Rarely, symptoms were reported at enrolment without an onset date. We imputed onset dates for these symptoms using the median number of days pre-enrolment (maximum 2 participants [0.55%] for rhinitis). TP = true positive, FN = false negative, TN = true negative, FP = false positive.

A - PCR-positive		Time Period (Days since exposure)											Time to Detection	
		0	1	2	3	4	5	6	7	8	9	10	%	Days
≥1 of the CS…	At Risk (TP + FN)	91	74	67	59	52	44	32	24	21	17	15	25	2
	Identified (TP)	17	7	8	6	4	7	6	3	4	1	1	50	6
	Lost	0	0	0	1	4	5	2	0	0	1	0	70	10
…OR sore throat	At Risk (TP + FN)	91	71	63	54	47	37	23	16	11	9	8	25	2
	Identified (TP)	20	8	9	6	7	9	5	5	2	0	0	50	5
	Lost	0	0	0	1	3	5	2	0	0	1	0	75	8
… OR headache	At Risk (TP + FN)	91	69	58	49	41	30	22	15	11	8	6	25	2
	Identified (TP)	22	11	9	7	8	4	5	4	3	1	0	50	4
	Lost	0	0	0	1	3	4	2	0	0	1	0	75	7
…OR muscle aches	At Risk (TP + FN)	91	73	64	54	46	38	27	20	18	14	13	25	2
	Identified (TP)	18	9	10	7	4	6	5	2	4	1	0	50	5
	Lost	0	0	0	1	4	5	2	0	0	0	0	75	9
…OR appetite loss	At Risk (TP + FN)	91	73	65	57	49	41	30	22	19	14	12	25	2
	Identified (TP)	18	8	8	7	4	6	6	3	5	1	0	50	6
	Lost	0	0	0	1	4	5	2	0	0	1	0	75	9
…or ≥1 of the EP	At Risk (TP + FN)	91	69	57	46	39	27	17	11	7	5	5	25	2
	Identified (TP)	22	12	11	6	9	6	4	4	2	0	0	50	4
	Lost	0	0	0	1	3	4	2	0	0	0	0	75	7
…or ≥2 of the EP	At Risk (TP + FN)	91	70	61	52	43	35	24	17	12	10	8	25	2
	Identified (TP)	21	9	9	8	5	6	5	5	2	1	0	50	4
	Lost	0	0	0	1	3	5	2	0	0	1	0	75	8
…or ≥3 of the EP	At Risk (TP + FN)	91	73	65	57	49	40	29	21	19	14	12	25	2
	Identified (TP)	18	8	8	7	5	6	6	2	5	1	0	50	5
	Lost	0	0	0	1	4	5	2	0	0	1	0	75	9
…or ≥4 of the EP	At Risk (TP + FN)	91	74	67	59	52	44	32	24	21	16	14	25	2
	Identified (TP)	17	7	8	6	4	7	6	3	5	1	0	50	6
	Lost	0	0	0	1	4	5	2	0	0	1	0	75	9

B - PCR-negative		Time Period (Days since exposure)											Time to Detection	
		0	1	2	3	4	5	6	7	8	9	10	%	Days
≥1 of the CS…	At Risk (TN + FP)	264	260	256	251	236	201	165	145	137	134	134	25	never
	Identified (FP)	4	4	5	8	6	3	1	2	1	0	0	50	never
	Lost	0	0	0	7	29	33	19	6	2	0	2	70	never
…OR sore throat	At Risk (TN + FP)	264	257	252	246	229	194	152	131	121	117	115	25	never
	Identified (FP)	7	5	6	10	7	10	3	4	2	2	0	50	never
	Lost	0	0	0	7	28	32	18	6	2	0	2	75	never
… OR headache	At Risk (TN + FP)	264	254	243	229	209	174	132	114	107	103	102	25	6
	Identified (FP)	10	11	14	14	10	11	5	2	2	1	0	50	never
	Lost	0	0	0	6	25	31	13	5	2	0	2	75	never
…OR muscle aches	At Risk (TN + FP)	264	258	253	247	230	195	158	138	131	128	128	25	never
	Identified (FP)	6	5	6	10	7	4	4	2	1	0	0	50	never
	Lost	0	0	0	7	28	33	16	5	2	0	2	75	never
…OR appetite loss	At Risk (TN + FP)	264	259	254	249	233	196	160	140	133	130	130	25	never
	Identified (FP)	5	5	5	9	8	5	2	2	1	0	0	50	never
	Lost	0	0	0	7	29	31	18	5	2	0	2	75	never
…or ≥1 of the EP	At Risk (TN + FP)	264	251	240	225	203	167	124	105	98	94	92	25	5
	Identified (FP)	13	11	15	16	13	14	6	3	2	2	0	50	never
	Lost	0	0	0	6	23	29	13	4	2	0	2	75	never
…or ≥2 of the EP	At Risk (TN + FP)	264	258	251	245	227	192	151	130	122	119	119	25	never
	Identified (FP)	6	7	6	11	6	9	6	3	1	0	0	50	never
	Lost	0	0	0	7	29	32	15	5	2	0	2	75	never
…or ≥3 of the EP	At Risk (TN + FP)	264	259	255	250	235	199	162	143	135	131	130	25	never
	Identified (FP)	5	4	5	8	7	4	1	2	2	1	0	50	never
	Lost	0	0	0	7	29	33	18	6	2	0	2	75	never
…or ≥4 of the EP	At Risk (TN + FP)	264	260	256	251	236	201	165	145	137	134	134	25	never
	Identified (FP)	4	4	5	8	6	3	1	2	1	0	0	50	never
	Lost	0	0	0	7	29	33	19	6	2	0	2	75	never

S15 (overleaf): Comparison of proposed symptom criteria with international case definitions. The presence or absence of symptoms by each day post-exposure was used to calculate diagnostic sensitivity (panel A) and specificity (panel B) for different case definitions (cohort D, figure 1) against a serial PCR reference standard. We compared the canonical symptoms (CS), our proposed symptom criteria (≥1 of the CS, or ≥2 of the EP) and international case definitions (S1, [main text references 8-12]). Where the precise wording of items in our symptom diaries (S2) did not correspond exactly with wording in international case definitions the closest approximation was chosen. In the WHO case definition, anorexia/nausea/vomiting is considered one symptom item whereas in our analysis loss of appetite and nausea/vomiting were considered two separate items, possibly overestimating sensitivity and underestimating specificity. In the US Centers for Disease Control case definition, chills and rigors are included as two separate symptom items as well as fever. In our analysis we considered fever as one variable, possibly underestimating sensitivity and overestimating specificity. The Clopper-Pearson interval was used to calculate 95% confidence intervals.

Kaplan-Meier plots were constructed showing time from exposure (index symptom onset for household-contacts) until positive identification by these symptom criteria in PCR-positive (panel C) and PCR-negative (panel D) contacts. The plot for the CS is shown in black. Rarely, symptoms were reported at enrolment without an onset date. We imputed onset dates for these symptoms by assuming the median number of days pre-enrolment (maximum 2 participants [0.55%] for rhinitis).

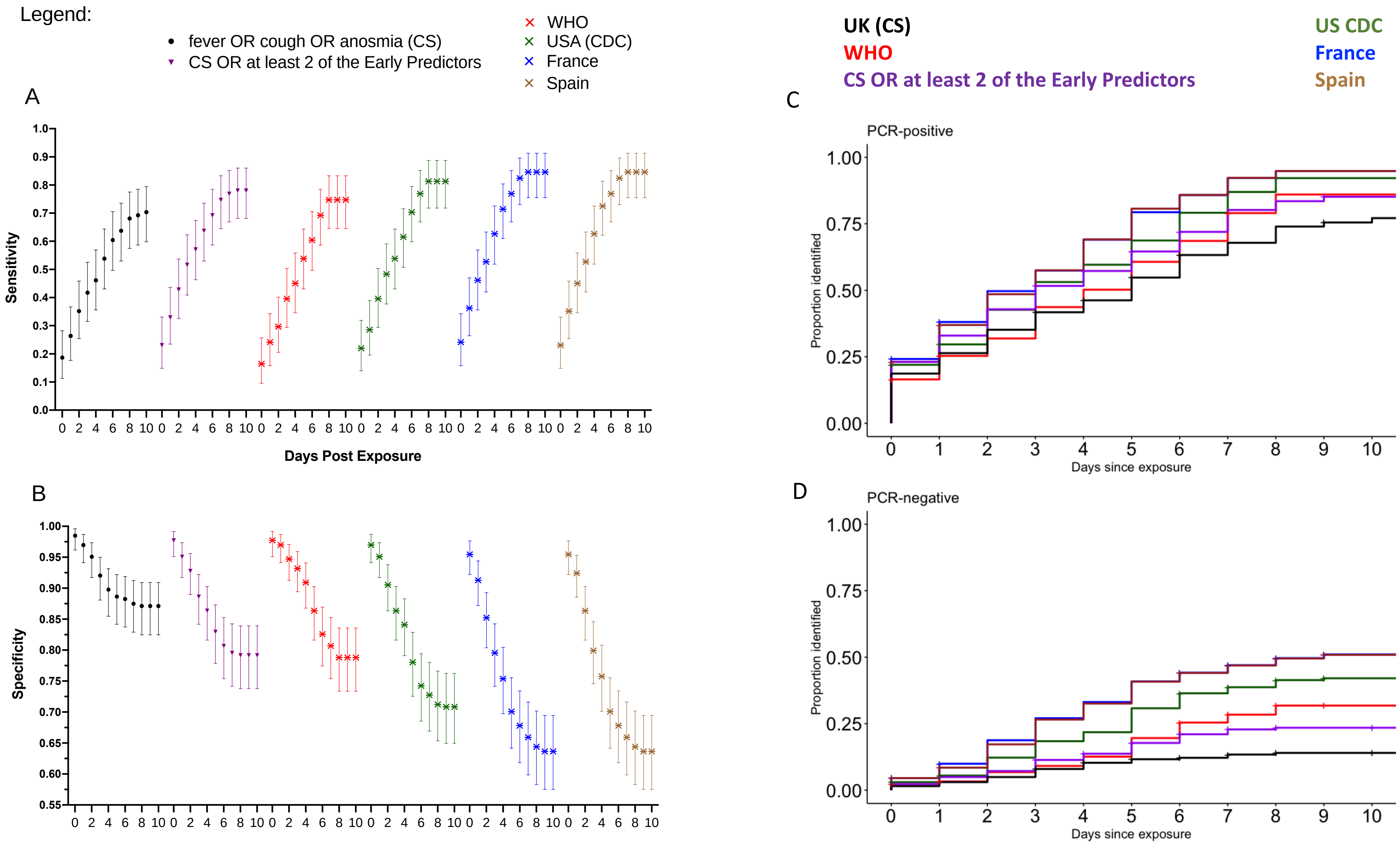

Legend:
fever OR cough OR anosmia (CS)
CS OR at least 2 of the Early Predictors
WHO
USA (CDC)
France
Spain
A
Sensitivity
Days Post Exposure
B
Specificity
UK (CS)
WHO
CS OR at least 2 of the Early Predictors
US CDC
France
Spain
C
PCR-positive
Proportion identified
Days since exposure
D
PCR-negative
Proportion identified
Days since exposure

1

S16: Leave-one-out analysis

Kaplan-Meier plots showing time from exposure until positive identification by different symptom criteria. The proportion of PCR-positive (left) and PCR-negative (right) participants who were positively identified by each case definition by each day following exposure (index symptom onset for household-contacts) is shown using a Kaplan-Meier plot. We compared the performance of the canonical symptoms (CS; fever OR cough OR anosmia; black line) with our proposed symptom criteria (≥1 of the CS, or ≥2 of the EP; purple line). These data are also shown in figure 5 (main text) and life-tables presented in S14.

We performed a leave-one-out analysis to determine whether each of the 'early predictor' (EP) symptoms (headache, muscle aches, sore throat, appetite loss) were necessary within the proposed symptom criteria. Four new symptom criteria (each with one of the EPs left out) are applied (red, green, blue and brown lines) and compared to the proposed symptom criteria. HA=Headache, MA=muscle aches, ST= sore throat, AL=appetite loss.

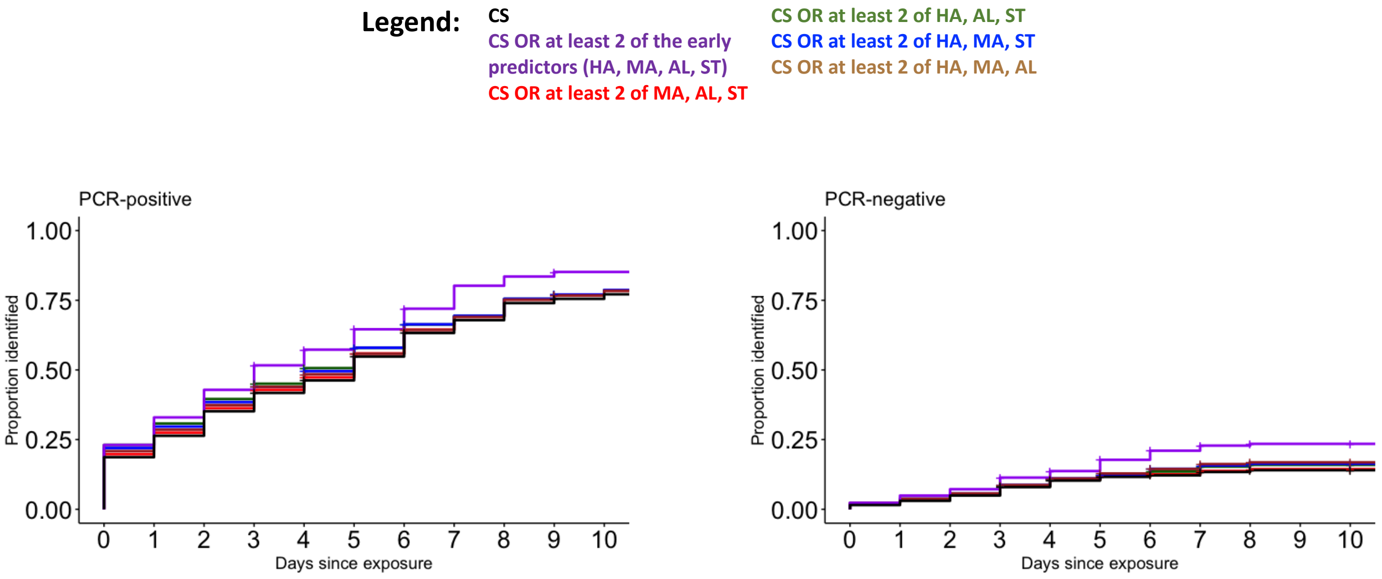

Supplement: Supplementary file 1 [file ERJ-02308-2021.Supplement.pdf]
