# Supplementary material for: Broadening symptom criteria improves early case identification in SARS-CoV-2 contacts
Source: Eur Respir J. 2022 Jul 7;60(1):2102308. doi: 10.1183/13993003.02308-2021 (PMC8620106; doi:10.1183/13993003.02308-2021)
Supplement: Supplementary file 2 [file ERJ-02308-2021.Shareable.pdf]

EUROPEAN RESPIRATORY JOURNAL
ORIGINAL RESEARCH ARTICLE
H. HOUSTON ET AL.

SHAREABLE PDF

Broadening symptom criteria improves early case identification in SARS-CoV-2 contacts

Hamish Houston[1,9], Seran Hakki[1,9], Timesh D. Pillay[1], Kieran Madon[1], Nieves Derqui-Fernandez[1], Aleksandra Koycheva[1], Anika Singanayagam[2], Joe Fenn[1], Rhia Kundu[1], Emily Conibear[1], Robert Varro[1], Jessica Cutajar[1], Valerie Quinn[1], Lulu Wang[1], Janakan S. Narean[1], Mica R. Tolosa-Wright[1], Jack Barnett[1], Onn Min Kon[1,3], Richard Tedder[4], Graham Taylor[5], Maria Zambon[2], Neil Ferguson[6], Jake Dunning[2,7], Jonathan J. Deeks[8,10] and Ajit Lalvani[1,10]

[1]NIHR Health Protection Research Unit in Respiratory Infections, Imperial College London, London, UK. [2]National Infection Service, Public Health England, London, UK. [3]Tuberculosis Service, Imperial College Healthcare NHS Trust, London, UK. [4]Molecular Diagnostics Unit, Imperial College London, London, UK. [5]Section of Virology, Dept of Infectious Disease, Imperial College London, London, UK. [6]Dept of Infectious Disease Epidemiology, Faculty of Medicine, Imperial College London, London, UK. [7]NIHR Health Protection Research Unit in Emerging and Zoonotic Infections, University of Oxford, Oxford, UK. [8]Test Evaluation Research Group, Institute of Applied Health Research, University of Birmingham, Birmingham, UK. [9]H. Houston and S. Hakki contributed equally. [10]J.J. Deeks and A. Lalvani contributed equally.

Corresponding author: Ajit Lalvani (a.lalvani@imperial.ac.uk)

Check for updates

Shareable abstract (@ERSpublications)
Data from two prospective longitudinal community-based cohorts show broadened symptom criteria identify more SARS-CoV-2 infections and reduce time to detection with minimal reductions in specificity, providing greater opportunities to prevent transmission. https://bit.ly/3wRpMeW

Cite this article as: Houston H, Hakki S, Pillay TD, *et al.* Broadening symptom criteria improves early case identification in SARS-CoV-2 contacts. *Eur Respir J* 2022; 60: 2102308 [DOI: 10.1183/13993003.02308-2021].

This single-page version can be shared freely online.

Copyright ©The authors 2022.

This version is distributed under the terms of the Creative Commons Attribution Licence 4.0.

Received: 22 Aug 2021
Accepted: 11 Nov 2021

Abstract

Background The success of case isolation and contact tracing for the control of severe acute respiratory syndrome coronavirus 2 (SARS-CoV-2) transmission depends on the accuracy and speed of case identification. We assessed whether inclusion of additional symptoms alongside three canonical symptoms (CS), *i.e.* fever, cough and loss or change in smell or taste, could improve case definitions and accelerate case identification in SARS-CoV-2 contacts.

Methods Two prospective longitudinal London (UK)-based cohorts of community SARS-CoV-2 contacts, recruited within 5 days of exposure, provided independent training and test datasets. Infected and uninfected contacts completed daily symptom diaries from the earliest possible time-points. Diagnostic information gained by adding symptoms to the CS was quantified using likelihood ratios and area under the receiver operating characteristic curve. Improvements in sensitivity and time to detection were compared with penalties in terms of specificity and number needed to test.

Results Of 529 contacts within two cohorts, 164 (31%) developed PCR-confirmed infection and 365 (69%) remained uninfected. In the training dataset (n=168), 29% of infected contacts did not report the CS. Four symptoms (sore throat, muscle aches, headache and appetite loss) were identified as early-predictors (EP) which added diagnostic value to the CS. The broadened symptom criterion “⩾1 of the CS, or ⩾2 of the EP” identified PCR-positive contacts in the test dataset on average 2 days earlier after exposure (p=0.07) than “⩾1 of the CS”, with only modest reduction in specificity (5.7%).

Conclusions Broadening symptom criteria to include individuals with at least two of muscle aches, headache, appetite loss and sore throat identifies more infections and reduces time to detection, providing greater opportunities to prevent SARS-CoV-2 transmission.
